# Target reliability-based design optimization studies on cohesive soil amended with chitosan and casein for liner applications

**DOI:** 10.1038/s41598-024-64408-7

**Published:** 2024-06-20

**Authors:** Romana Mariyam Rasheed, Arif Ali Baig Moghal, B. Munwar Basha, Abdullah Almajed

**Affiliations:** 1 Department of Civil Engineering, TKM College of Engineering, Kollam, 691005 India; 2 Department of Civil Engineering, National Institute of Technology Warangal, Warangal, 506004 India; 3 Department of Civil Engineering, Indian Institute of Technology Hyderabad, Hyderabad, 502284 India; 4 Department of Civil Engineering, College of Engineering, King Saud University, 11421 Riyadh, Saudi Arabia

**Keywords:** Civil engineering, Environmental sciences

## Abstract

The current study investigated the primary and secondary compressibility characteristics of organic clay with two biopolymers, Chitosan (*D*_*ch*_) and Casein (*D*_*ca*_) at dosages of 0.5%, 1%, 2%, and 4%. The primary compression index (*C*_*c*_) values were reduced by 18% and 59% at dosage (*D*_*ch*_ and *D*_*ca*_) of 4% at a consolidation pressure of 800 kPa. The secondary compression indices of chitosan and casein-treated soils fell below the normal range specified for organic soils and lay in the range of 0.01–0.017. The biopolymers also accelerated the consolidation process at all dosages (*D*_*ch*_) and 2% *D*_*ca*_. The hydraulic conductivity increased for all dosages of chitosan whereas it declined for all dosages of casein compared to untreated soil. The reliability analysis was conducted for biopolymer-treated soils and presented a rational approach toward the selection of a suitable liner. Chitosan failed to achieve a target reliability index of 3 whereas casein-amended samples attained values equal to and greater than 3 at all dosages and consolidation pressures at *COV of K*_*max*_ = 20%. At all dosages, the casein-treated soils exhibited reliability index values greater than 3 up to *COV of K*_*max*_ = 40% indicating the higher stability of casein mixes as a liner material.

## Introduction

Since time immemorial, waste disposal and management have been a pertinent issue for mankind. The situation is aggravated by rapid urbanization and dwindling waste processing^1^. The major environmental issue faced by geoenvironmental engineers is the potential contamination of groundwater and surrounding ecosystems by the leachate produced by solid waste^2^. The contaminant transport and the associated mechanisms within soil and water pose a significant threat and are key issues to be addressed^3^. Retardation of contaminants within the landfill facility is the immediate and promising solution to delaying the environmental contamination from municipal solid waste (MSW) landfills. Landfill liners form an integral part of the waste management facilities by retarding the migration of organic and inorganic contaminants^4,5^. The material serving as a liner has to comply with the design requirements by satisfying the required values of unconfined compressive strength (200 kPa), hydraulic conductivity (< 10^−7^ cm/s), and volumetric shrinkage (< 4%)^6,7^.

Locally available soils may not always satisfy the liner design specifications. The presence of vulnerable soils as in situ soils poses a threat to the structural integrity and serviceability of the intended application^8^. However, the amendment of soils using appropriate materials will render them effective barriers^9^. Partial or complete replacement of local soil by bentonite can significantly modify properties such as hydraulic conductivity, consistency, and compaction characteristics^10^. However, the higher shrinkage, low compressive strength, and high-cost render bentonite an unreliable liner material^11^. In the past, industrial waste products such as fly ash have also been proposed as barrier materials owing to their significantly better mechanical and hydraulic properties^12^. Regardless of their benefits, fly ash has proven to be unsuitable in field application owing to the difficulty in compacting, leading to higher hydraulic conductivities^13^. The environmental suitability of fly ash is also questionable due to the presence of heavy metals^13^. The inclusion of silica fume (10–25%) in clayey soil led to increased cation exchange capacity and a reduction in hydraulic conductivity. The percentage removal of copper was achieved around 90% with silica fume at 25% dosage^14^. Due to the disposal challenge posed by waste tire textile fibers (WTTF), these materials were utilized in expansive soils to investigate the consolidation, desiccation cracking, and tensile strength. The efficacy of soil-WTTF mixes as a suitable liner material was proved by the reduction in compressibility and desiccation cracking^15^.

The structural integrity of compacted clay liners is sometimes compromised by the rainfall infiltration caused by desiccation cracking. Under this phenomenon, the hydraulic conductivity increased by manifold and affected the liner functionality^16^. Chitosan has effectively controlled the desiccation cracking in fine-grained soils for dosages of 0.5–4%^17^. Steadily, a paradigm shift has been witnessed from the usage of chemical amendments to novel materials in recent years^18–23^. Hydrophobic and waste-derived biopolymers such as chitosan have proven their worth in amending the soil to suit liner requirements at optimal dosages^17,23^. By natural selection, these materials tend to be resilient and relatively sustainable compared to other chemical stabilizers^24^. The filler characteristics of chitosan have modified the soil structure to have lower hydraulic conductivities (< 10^−7^ cm/s) at lower dosages (< 2%) in organic silts. Additionally, the compression index was reduced by 50% at 0.5% chitosan^25^. Another emerging biopolymer in the field of soft soil stabilization is a protein-based biopolymer named casein. The casein structure facilitates reduced interactions with water molecules due to the presence of hydrophobic bonds inside the casein micelles and aids in reducing the compressibility of the soil by 71% at a dosage of 2% in clayey soil^26^.

It is important to understand that biopolymers are degradable and will be subjected to changes in the measured properties upon contact with the heavy metals and leachate produced by the landfill. The hydraulic conductivity (*K*) of the biopolymer amended liner is subjected to variations due to factors such as construction techniques, overburden pressure from waste, rainfall infiltration, and interaction with other organic matter. The dosages selected for liner amendment will have a profound impact on the hydraulic characteristics of the material. It becomes imperative to evaluate the safety of the liners against *K* failure by conducting a reliability-based design optimization (RBDO). The reliability analysis is useful in handling complex information such as multivariate correlated data and leads to sound engineering judgment by verifying the reasonability of results^9,27–29^. This rational approach is beneficial to providing a comparison of probabilities of failure for different designs and also demonstrates the role of different components to uncertainty in the probability of failure^27,30–33^.

The current study investigated the possibility of using a Silty Clay amended by including a polysaccharide (chitosan) and protein-based (casein) biopolymer by experimental data, multivariate statistical models, and RBDO. The manuscript also discusses the mechanism between soil and the selected biopolymers leading to the desired requirements. The initial part of the manuscript discusses the results from an incremental consolidation test performed on various soil-biopolymer mixes (*D*_*ch*_ and *D*_*ca*_) leading to the selection of an optimal mix. The reliability of the optimal mixes was determined by performing RBDO and evaluation of reliability indices.

## Materials and methodology

### Soil

The soil sample was procured from Meenapally, Kuttanad, India (9° 50.9′ 50.2″ N, 76° 39′ 40.28″ E) at a depth of 1.5 m. The organic content was obtained as 13% as per AASHTO T 267^34^. The liquid limit and plasticity index values under air-dried conditions were determined as 73% and 29.4%, respectively, by ASTM D4318-17e1 (ASTM 2017)^35^. The Maximum Dry Density and Optimum Moisture Content were determined as 1.48 g/cm^3^ and 31.1%, respectively, as per ASTM D698-12e2 (ASTM 2021)^36^. The soil can be classified as organic clay of high plasticity as per ASTM D2487-17 (ASTM 2020)^37^. Further, the one-dimensional incremental consolidation test was conducted by following ASTM D2435-04 (ASTM 2011)^38^ to assess the compressibility characteristics.

### Biopolymers

The biopolymers considered for the current study were chitosan and casein. The aforementioned biopolymers were procured from Swakit Biotech Private Limited, Karnataka, India, and Marine Hydrocolloids, Cochin, Kerala, India. Owing to their hydrophobic properties and significant improvement in engineering properties, the biopolymers were considered for amendment at dosages (*D*_*ch*_ and *D*_*ca*_) of 0.5%, 1%, 2%, and 4%. The dosages were fixed considering the previous studies of Chitosan and Casein on cohesive soils with different mineralogy and composition^25,39–41^. The dosages were limited to 4% as any further increase in dosage resulted in increased viscosity of the biopolymer gels formed due to interaction with water, thereby affecting the bonding between soil and biopolymer^39^. The high viscosity of the biopolymer gels will lead to poor workability and accelerate the formation of air voids resulting in weak planes^21^. Owing to the scarcity of studies on organic soils amended using biopolymers, the influence of biopolymers, and particularly the effect of chitosan and casein on cohesive soils were referred for understanding the range of dosages to be selected for the current study.

### One-dimensional consolidation test

The raw and biopolymer-amended soils were evaluated for their compressibility characteristics by conducting a conventional one-dimensional fixed ring consolidation test following ASTM D2435-04 (ASTM 2011)^38^. The samples were prepared in the consolidation ring of dimension 60 mm × 20 mm at a maximum dry density of 1.48 g/cm^3^. Following the sample preparation within the consolidation ring, sample saturation was initiated under a seating pressure of 6.25 kPa. The consolidation test was conducted with a load increment up to 800 kPa followed by unloading at a decrement ratio of four. Based on the experimental data, the parameters such as coefficient of consolidation (*C*_*v*_), hydraulic conductivity (*K*), primary compression index (*C*_*c*_), and secondary compression index (*C*_*α*_) were calculated at consolidation pressures ($\sigma_{cp}$) of 100, 200, 400, and 800 kPa. From the previous experimental studies, it was observed that the completion time for primary and secondary consolidation of organic soils is exponentially related to the load or stress applied^42^. Furthermore, when soils are ameliorated using biopolymers or such organic materials, the compressibility behaviour will deviate from that of untreated soil^21^. It was observed that above a load of 400 kPa, the secondary consolidation behaviour does not change much for soils with organic content in the range of 10–50%. In the initial studies conducted on the collected soil, there was negligible variation in deformation observed beyond 800 kPa. Additionally, the compressibility behaviour of Kaolinite cohesive soils modified using biopolymers compacted at maximum dry density was also conducted for the pressure range of 12.5–800 kPa^43–45^. Hence the stress range for the current study was limited to 800 kPa. The tests were conducted in triplicates and the average values were considered for analysis.

### Multivariate nonlinear regression models for consolidation parameters

The parameters derived from the consolidation data such as the primary compression index (*C*_*c*_), secondary compression index (*C*_*α*_), coefficient of consolidation (*C*_*v*_), and hydraulic conductivity (*K*) of organic clay treated with Chitosan (*D*_*ch*_) and Casein (*D*_*ca*_) were modelled using twelve non-linear regression models. The statistical software package (DataFit, Oakdale Engineering, PA), which contains 298 2D and 242 3D regression models was utilized to develop the multivariate regression equations. The independent variables considered are dosages of Chitosan (*D*_*ch*_) and Casein (*D*_*ca*_), and consolidation pressure ($\sigma_{cp}$). From the statistical analysis of the experimental data, it was observed that the non-linear regression models predicted the behaviour accurately compared to linear models. The models having the highest coefficient of determination (*R*^2^) and lower root mean square error (RMSE) were selected as the best-fitting non-linear relationships. A 95% confidence was used to evaluate the regression coefficients. The probability of liner material failure, the factors of safety, and the limit state function for the hydraulic conductivity failure with unamended organic clay are given in Eqs. 1, 2, and 3 respectively.

The appendix presents the multivariate non-linear regression models developed for untreated and biopolymer-treated organic clay for predicting the values of primary compression index (*C*_*c*_), secondary compression index (*C*_*α*_), coefficient of consolidation (*C*_*v*_), and hydraulic conductivity (*K*). The nonlinear regression models for primary compression indices of untreated organic clay ($C_{c\_ut\_fit}$), organic clay amended with Chitosan ($C_{c\_Chi\_fit}$), and Casein ($C_{c\_Ca\_fit}$) are displayed in Eqs. 4, 5, and 6. The equations (7, 8, and 9) are determined to predict secondary compression indices of untreated organic clay ($C_{\alpha \_ut\_fit}$), treated with Chitosan ($C_{\alpha \_Chi\_fit}$), and Casein ($C_{\alpha \_Ca\_fit}$). The Appendix also presents the equations (10, 11, and 12) for coefficients of consolidation of untreated organic clay ($C_{v\_ut\_fit}$), treated with Chitosan ($C_{v\_Chi\_fit}$), and Casein ($C_{v\_Ca\_fit}$). Lastly, the equations (13, 14, and 15) to predict the hydraulic conductivity ($K_{ut\_fit}$) of untreated organic clay, treated with Chitosan ($K_{Chi\_fit}$), and Casein ($K_{Ca\_fit}$) are shown in Appendix.

Tables S1 to S12 summarized the non-linear regression analysis of the primary compression index (*C*_*c*_), secondary compression index (*C*_*α*_), coefficient of consolidation (*C*_*v*_), and hydraulic conductivity (*K*) for organic clay, Chitosan (*D*_*ch*_), and Casein (*D*_*ca*_) treated organic clay under supplementary material. Tables S1 to S12 established a good correlation between experimental findings and values proposed using the multivariate regression equations. The effect of adding Chitosan content (*D*_*ch*_) and Casein content (*D*_*ca*_) to organic clay on the factors of safety ($FS_{{D_{ch} }}$ and $FS_{{D_{ca} }}$) and reliability indices against liner material failure ($\beta_{Dch}$ and $\beta_{Dca}$) was discussed in the results and discussion section.

### Reliability-based design optimization of liner material for MSW landfills

The reliability-based design optimization (RBDO) approach was utilized to assess the integrity of MSW liners against the possibility of hydraulic conductivity failure. The ideal value of hydraulic conductivity of liners is considered to be less than 10^−7^ cm/s. The probability of liner material failure (*P*_*f*_) in terms of hydraulic conductivity (*K*) failure is defined in Eq. (1):

1 $$P_{f} = P\left( {K_{\max } < K} \right)$$

The limiting value of hydraulic conductivity defined to prevent failure is *K*_*max*_. To ensure serviceability of liners, *K*_*max*_ is considered as 10^−8^ cm/s. The factors of safety against hydraulic conductivity failure of unamended organic clay ($FS$) is given by Eq. (2).

2 $$FS = \frac{{K_{\max } }}{{K_{fit} }}$$

The factors of safety against hydraulic conductivity failure of chitosan-treated organic clay ($FS_{{D_{ch} }}$), and casein-treated organic clay ($FS_{{D_{ca} }}$) can be computed by substituting $K_{fit}$ = $K_{{D_{ch} \_fit}}$, and $K_{fit}$ = $K_{{D_{ca} \_fit}}$ in Eq. (2).

The limit state function for the hydraulic conductivity failure of liner material with unamended organic clay is given by Eq. (3).

3 $$g\left( x \right) = FS - 1$$

Similarly, limit state functions for the hydraulic conductivity failure of liner material with Chitosan treated soil ($g_{1} \left( x \right)$) and Casein treated soil ($g_{2} \left( x \right)$) can be written by substituting $FS$ = $FS_{{D_{ch} }}$ and $FS$ = $FS_{{D_{ca} }}$ in Eq. (3).

The optimization in the standard normal space $U\, = \,\left\{ {u_{k} } \right\}_{k = 1}^{n}$ is defined as follows:

- Determine the reliability index against hydraulic conductivity failure ($\beta_{Dch}$) of Chitosan-treated liner material, which minimizes $g_{1} (u)$ and is subjected to $\sqrt {u^{T} u}$.
- Determine the reliability index against hydraulic conductivity failure ($\beta_{Dca}$) of Casein-treated liner material which minimizes $g_{2} (u)$ and is subjected to $\sqrt {u^{T} u}$.

The conditions, $g_{1} (u) = 0$ and $g_{2} (u) = 0$ represent the linearized first-order limit state functions in the U space related to the risk of hydraulic conductivity failure in liner material made of organic clay treated with Chitosan and Casein, respectively.

### Target reliability index for RBDO analysis

The target reliability levels used in geotechnical and geoenvironmental engineering designs are influenced by guidelines from various national and international codes of practice. However, these recommendations are not always consistent across different standards^46^. The selection of an appropriate target reliability index balances safety and economic considerations, and specific values are often chosen based on project requirements and codal provisions. A target reliability index of 3.0 for above-average performance, 4.0 for good performance, and 5.0 for high performance is recommended by the US Army Corps of Engineers^47^. Whereas, Eurocode 7 recommends a target reliability level of 95%^48^. The target reliability index is chosen to meet codal provisions and to ensure safety while also considering economic factors. The design of liners for high-reliability index implies a very safe design but may lead to conservative and costly solutions. The design of liners for low-reliability index may result in an unsafe design, though it could be less expensive. A reliability index of 3.0 corresponds to a probability of failure of approximately 0.00135 (or 0.135%) is commonly prescribed in geotechnical and geoenvironmental design practices^49^. This level of risk is deemed acceptable for many geotechnical engineering applications where the consequences of failure are significant but not catastrophic. This value is seen as a rational compromise between safety and cost. The importance of selecting project-specific target reliability indices has been emphasized by researchers, as different projects may have varying requirements for safety and cost-effectiveness^50^. The target reliability index is particularly critical in designs such as landfill liners, where a high-reliability index ensures safety but can be conservative and costly. Conversely, a lower reliability index might reduce costs but at the risk of compromising safety. Therefore, a target reliability index of 3.0 is chosen in the present study as it is a well-established benchmark that balances safety, cost, and practicality.

## Results and discussions

### Variation in void ratio with consolidation pressure

**Figure 1:**
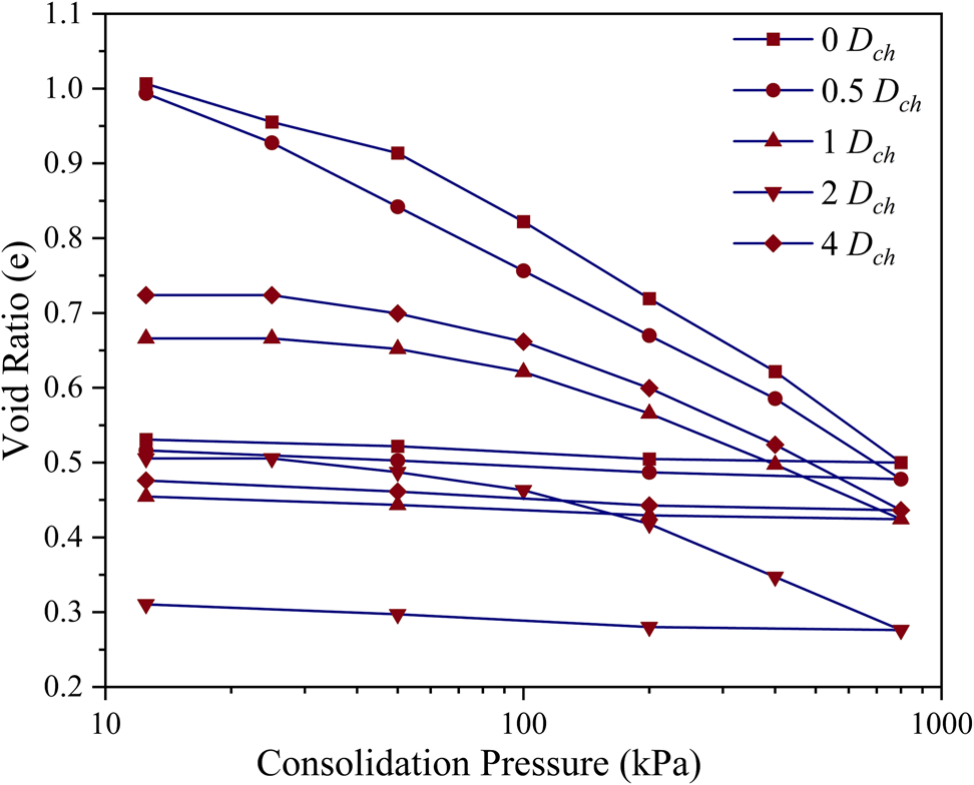
Variation in void ratio with consolidation pressure for chitosan modified soil.

**Figure 2:**
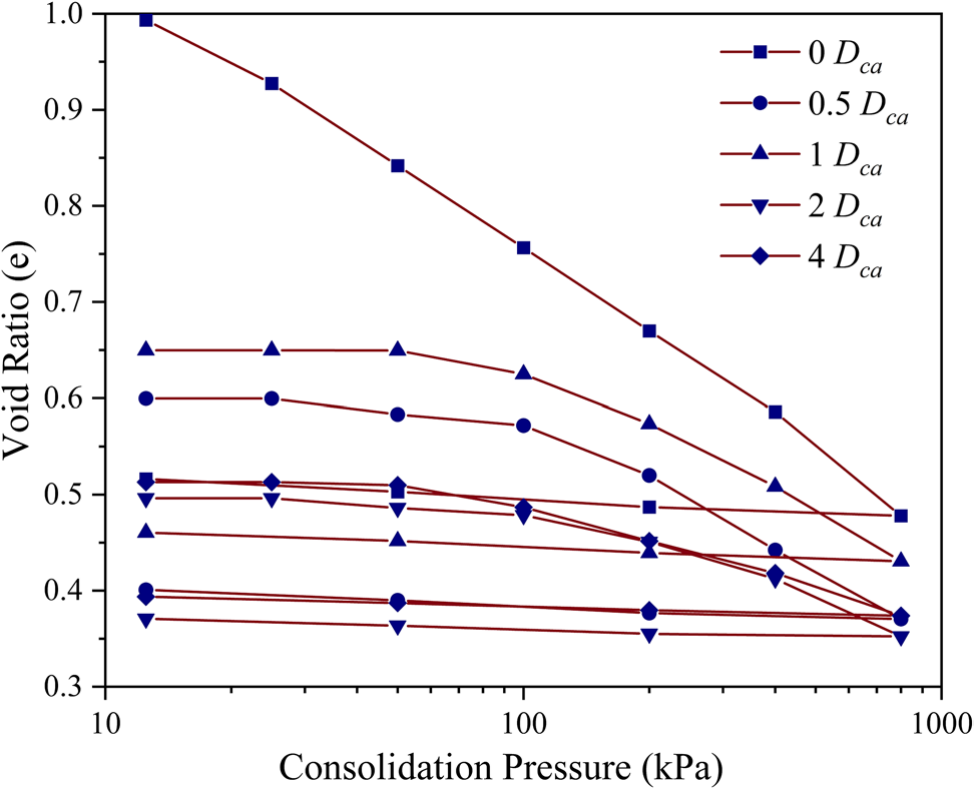
Variation in void ratio with consolidation pressure for casein modified soil.

**Figure 3:**
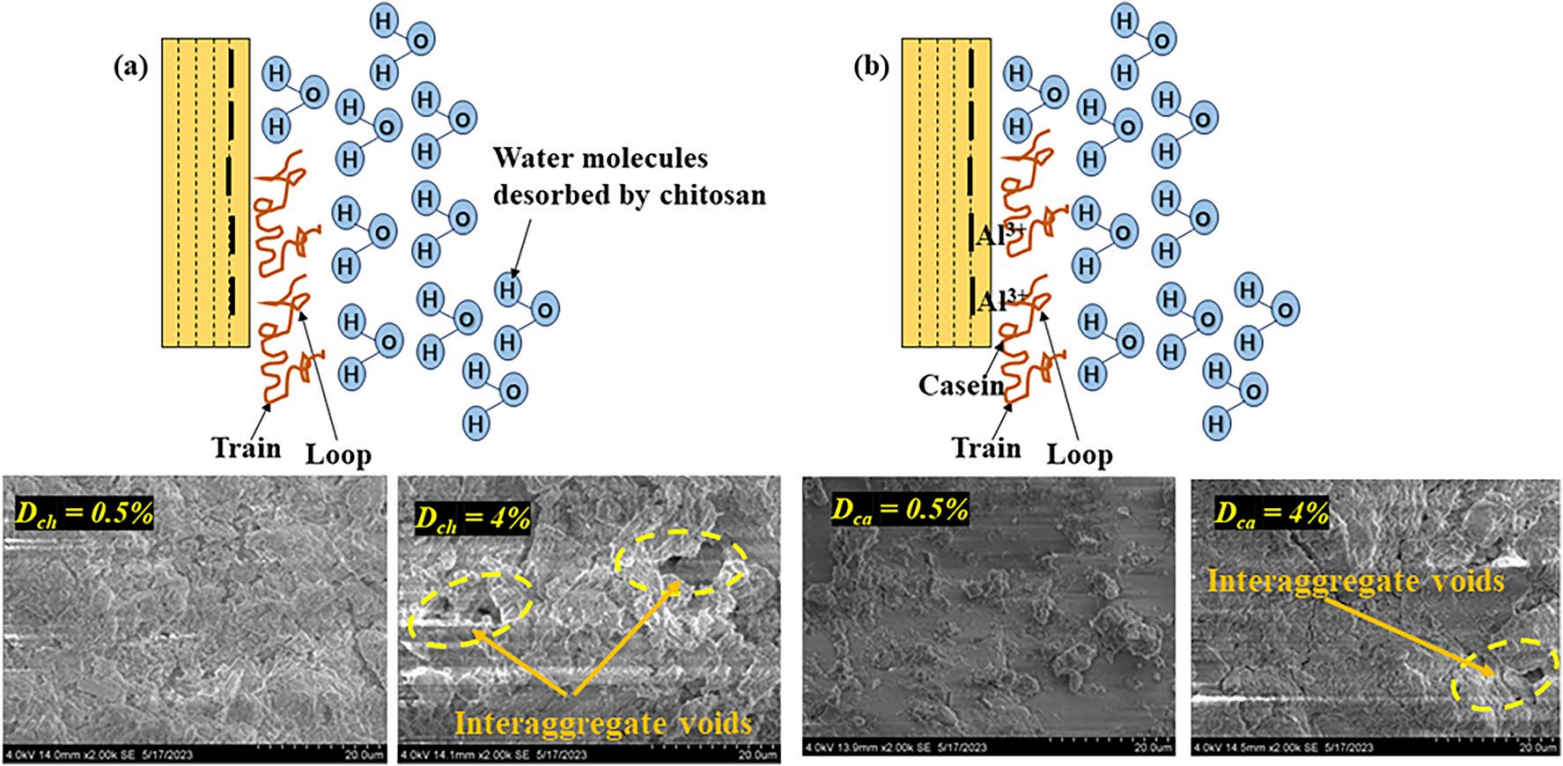
Mechanism of interaction between soil and (**a**) Chitosan, (**b**) Casein.

As depicted in Fig. 1, the untreated soil exhibited a marginal decrease in void ratio up to a pressure of 50 kPa beyond which the void ratio sustained a steep decrease with an increase in pressure up to 800 kPa. Upon unloading at 800 kPa, the rebound was found to be marginal indicating the presence of Kaolinite mineral in the studied soil. As shown in Fig. 1, the inclusion of chitosan led to a reduction in the void ratio at all dosages and consolidation pressures. At a consolidation pressure of 100 kPa, the void ratio decreased by 10.6%, 23.5%, 42.4%, and 18.8% at 0.5%, 1%, 2%, and 4% *D*_*ch*_ respectively. The soil modified using casein also exhibited a similar decrease in void ratio and was reduced by 30.5%, 24.7%, 43.5%, and 42.3% at 0.5%, 1%, 2%, and 4% *D*_*ca*_ respectively as shown in Fig. 2. For the same dosages, it can be observed from Figs. 1 and 2 that casein overpowered chitosan in reducing the compressibility at all dosages. The chitosan and casein-treated soils exhibited a marginal rebound upon unloading. The chitosan and casein modified the soil structure to a flocculated and aggregated structure as shown in Fig. 3. Casein-treated soils exhibited a denser structure compared to chitosan-treated soil and this was also reflected in the compressibility of the treated soils. The steep decrease in void ratio beyond a pressure of 100 kPa indicated the disruption of the stable structures formed as a result of soil-biopolymer bonding. For chitosan and casein-treated soils, it was observed that the void ratio increased at 4% dosage compared to the void ratios at lower dosages indicating the formation of higher interaggregate voids within the structure as depicted in Fig. 3.

### Variation in primary compression index (***C***_***c***_) and secondary compression index (***C***_***α***_)

**Figure 4:**
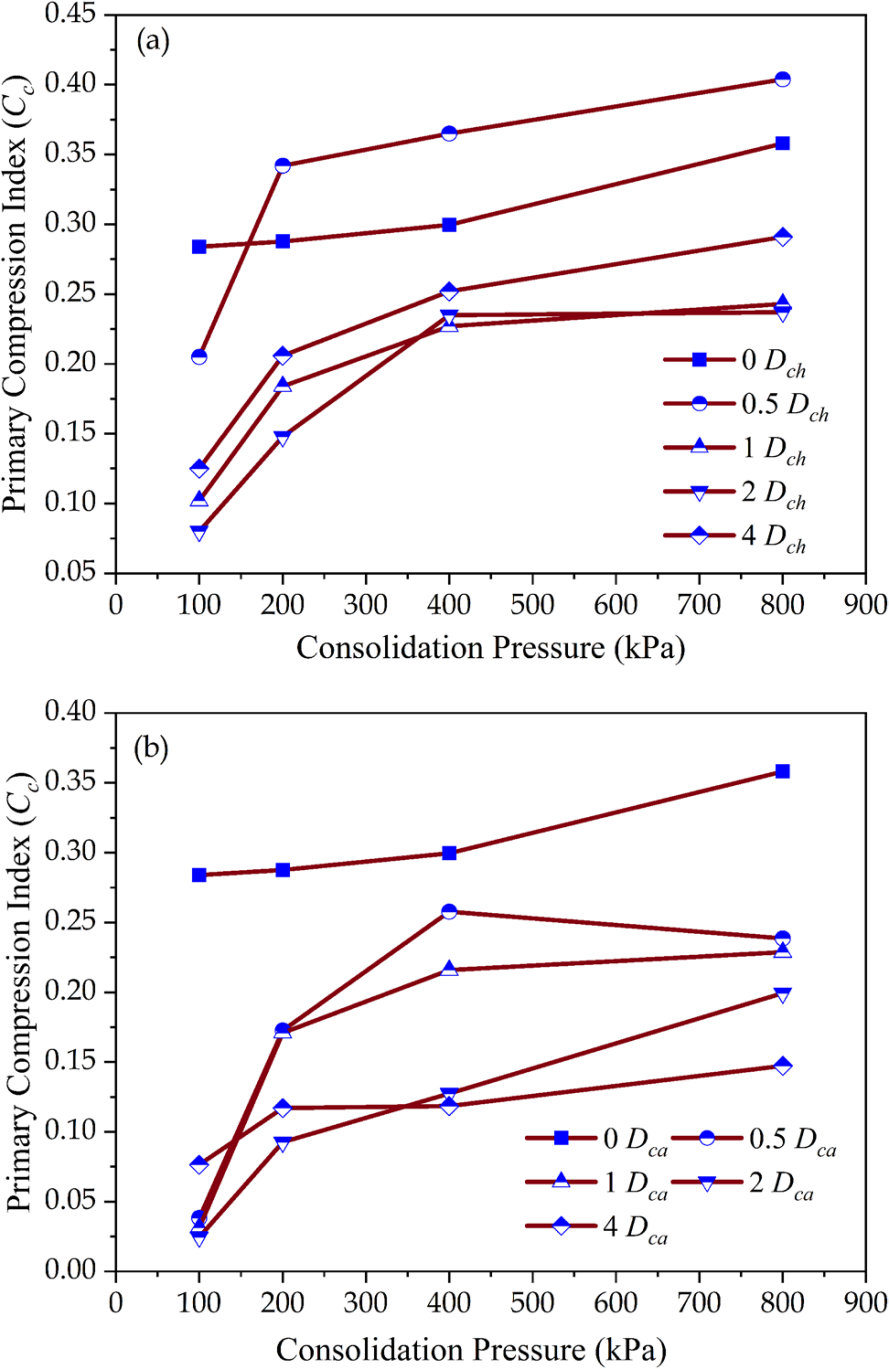
Variation of primary compression index (*C*_*c*_) values (**a**) Chitosan modified soil, (**b**) Casein modified soil.

The effect of dosage and consolidation pressure on the primary compression index (*C*_*c*_) is depicted in Fig. 4a. The untreated soils exhibited *C*_*c*_ of 0.284, 0.289, 0.299, and 0.358 at consolidation pressure of 100, 200, 400, and 800 kPa respectively. The addition of chitosan reduced the volume of the soil matrix by the interaction between the chitosan and soil particles. The neutrally charged chitosan particles replaced the water molecules existing near the clay surface and reduced the void space between the soil particles. The soil matrix transformed from flocculated to flocculated and aggregated structure and thereby reduced the volume of the soil-biopolymer matrix. This led to the reduction in the primary compression index (*C*_*c*_) by 0.72, 0.35, 0.27, and 0.35 times at *D*_*ch*_ = 0.5%, 1%, 2%, and 4% respectively at a consolidation pressure of 100 kPa. When the consolidation pressure was increased to 800 kPa, the disruption of the stable matrices increased the compressibility and attained higher values of *C*_*c*_ at a particular dosage (*D*_*ch*_). The inclusion of casein micelles led to an interaction between the negatively charged casein and positively charged edges of clay particles leading to a uniform coating around the soil particles. The casein coating reduced the compressibility more effectively compared to chitosan particles. At a consolidation pressure of 100 kPa, the compressibility (*C*_*c*_) reduced by 0.13, 0.11, 0.08, and 0.21 times compared to untreated soil at dosages of *D*_*ca*_ = 0.5%, 1%, 2%, and 4% respectively as shown in Fig. 4b.

**Figure 5:**
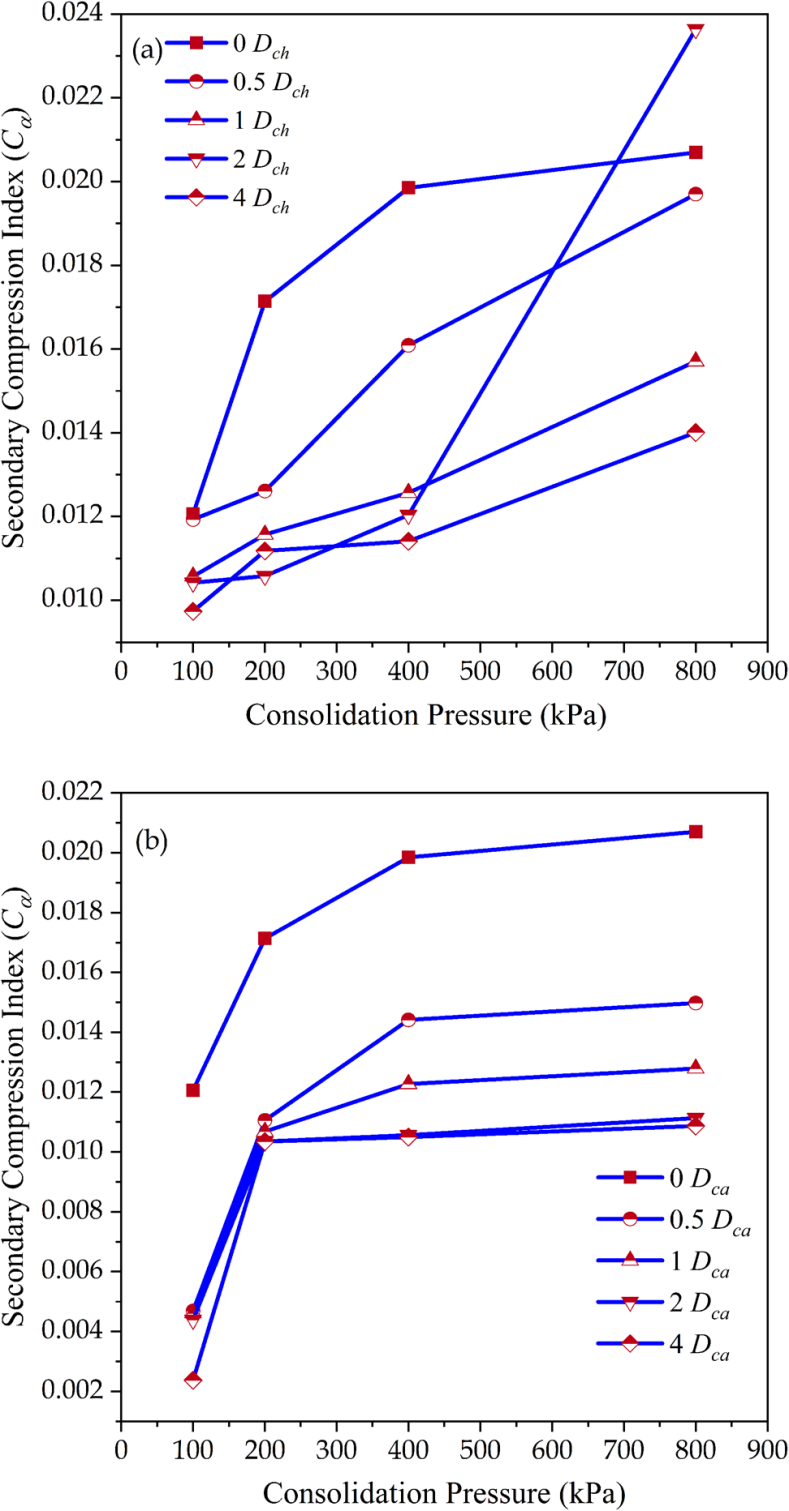
Variation of secondary compression index (*C*_*α*_) values (**a**) Chitosan modified soil, (**b**) Casein modified soil.

The effect of chitosan and casein in reducing the creep deformation is depicted in Fig. 5a and b. The raw soil exhibited secondary compression index (*C*_*α*_) values of 0.0121, 0.0171, 0.0199, and 0.021 at $\sigma_{cp}$ = 100, 200, 400, and 800 kPa respectively. Soil with *C*_*α*_ greater than or equal to 0.064 is considered to be highly organic^39^. The studied soil exhibited low values of *C*_*α*_ around 0.01 due to the lower organic content of the soil. At $\sigma_{cp}$ = 100 kPa, the chitosan-treated soil reduced *C*_*α*_ by 1.07%, 12.35%, 13.67%, and 19.24% for dosages of (*D*_*ch*_) 0.5%, 1%, 2%, and 4% respectively. Whereas, the casein-treated soil reduced *C*_*α*_ by 61.11%, 61.77%, 63.59%, and 80.35% for dosages (*D*_*ca*_) of 0.5%, 1%, 2%, and 4% respectively. The reduction in secondary compression after amendment with chitosan and casein will aid in mitigating the detrimental effects of secondary settlement in the field.

### Variations in coefficient of consolidation (***C***_***v***_)

**Figure 6:**
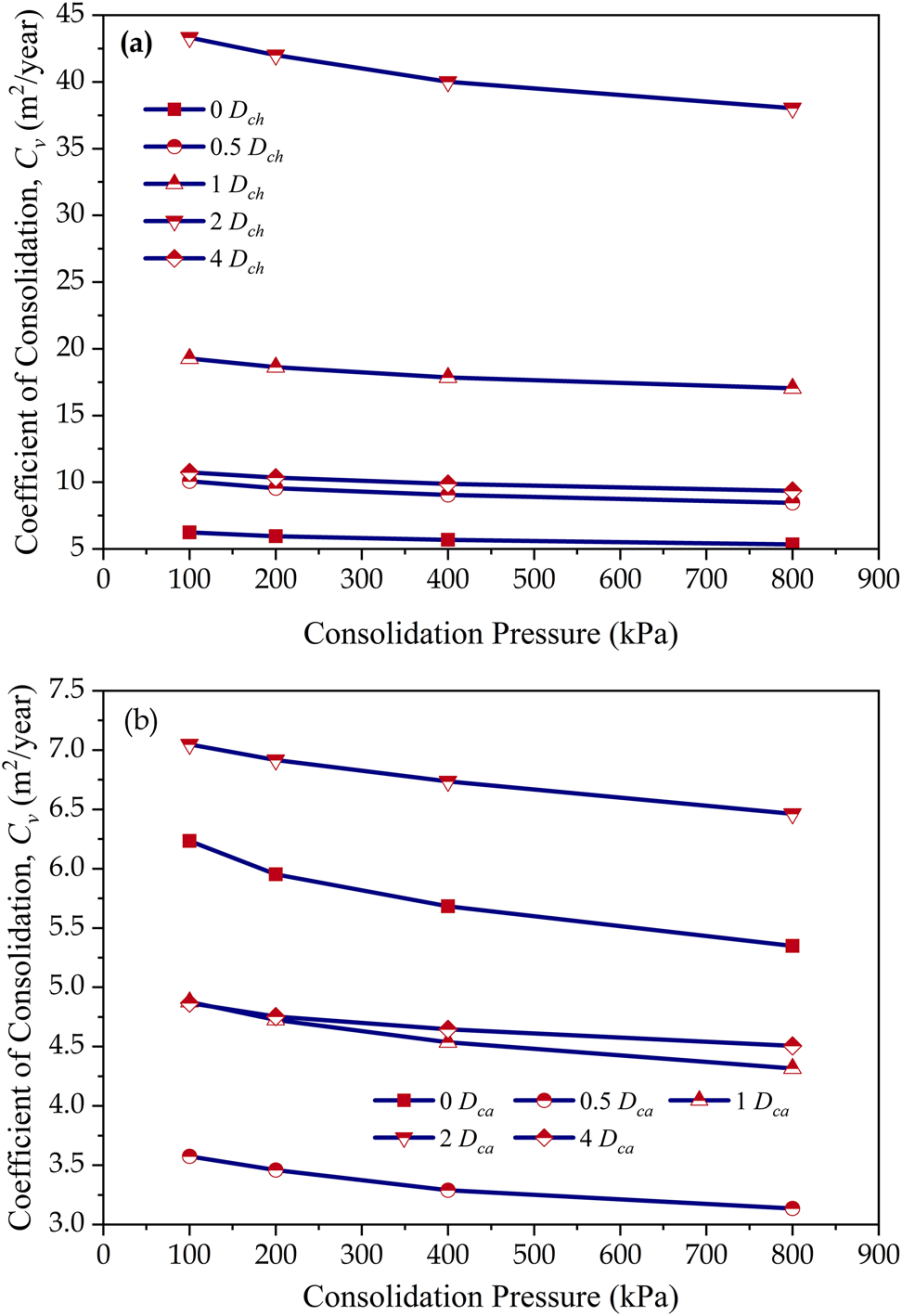
Variation of coefficient of consolidation (*C*_*v*_) values (**a**) Chitosan modified soil, (**b**) Casein modified soil.

The variation of *C*_*v*_ in chitosan-treated (*D*_*ch*_) soils is presented in Fig. 6a. The untreated soils attained *C*_*v*_ values of 6.24, 5.95, 5.68, and 5.34 m^2^/year at consolidation pressures of 100 kPa, 200 kPa, 400 kPa, and 800 kPa respectively. From Fig. 6a, it was observed that the addition of chitosan (*D*_*ch*_) accelerated the consolidation process and facilitated the easy expulsion of water from the soil structure at all dosages (*D*_*ch*_). At a consolidation pressure of 100 kPa, the *C*_*v*_ values increased by 1.62, 3.09, 6.94, and 1.72 times at *D*_*ch*_ = 0.5%, 1%, 2%, and 4% respectively. For a particular consolidation pressure, the *C*_*v*_ values increased up to 2% and thereafter declined at *D*_*ch*_ = 4%. The casein-treated (*D*_*ca*_) soils exhibited a drop in *C*_*v*_ for all dosages except for *D*_*ca*_ = 2%. At a consolidation pressure of 100 kPa, the *C*_*v*_ values dropped by 0.57, 0.78, and 0.78 times for dosages of *D*_*ca*_ = 0.5%, 1%, and 4% as shown in Fig. 6b. Similar to the trend observed for chitosan, the casein-treated soil also displayed a decrement in *C*_*v*_ with a rise in consolidation pressure.

### Variation in hydraulic conductivity (*K*)

**Figure 7:**
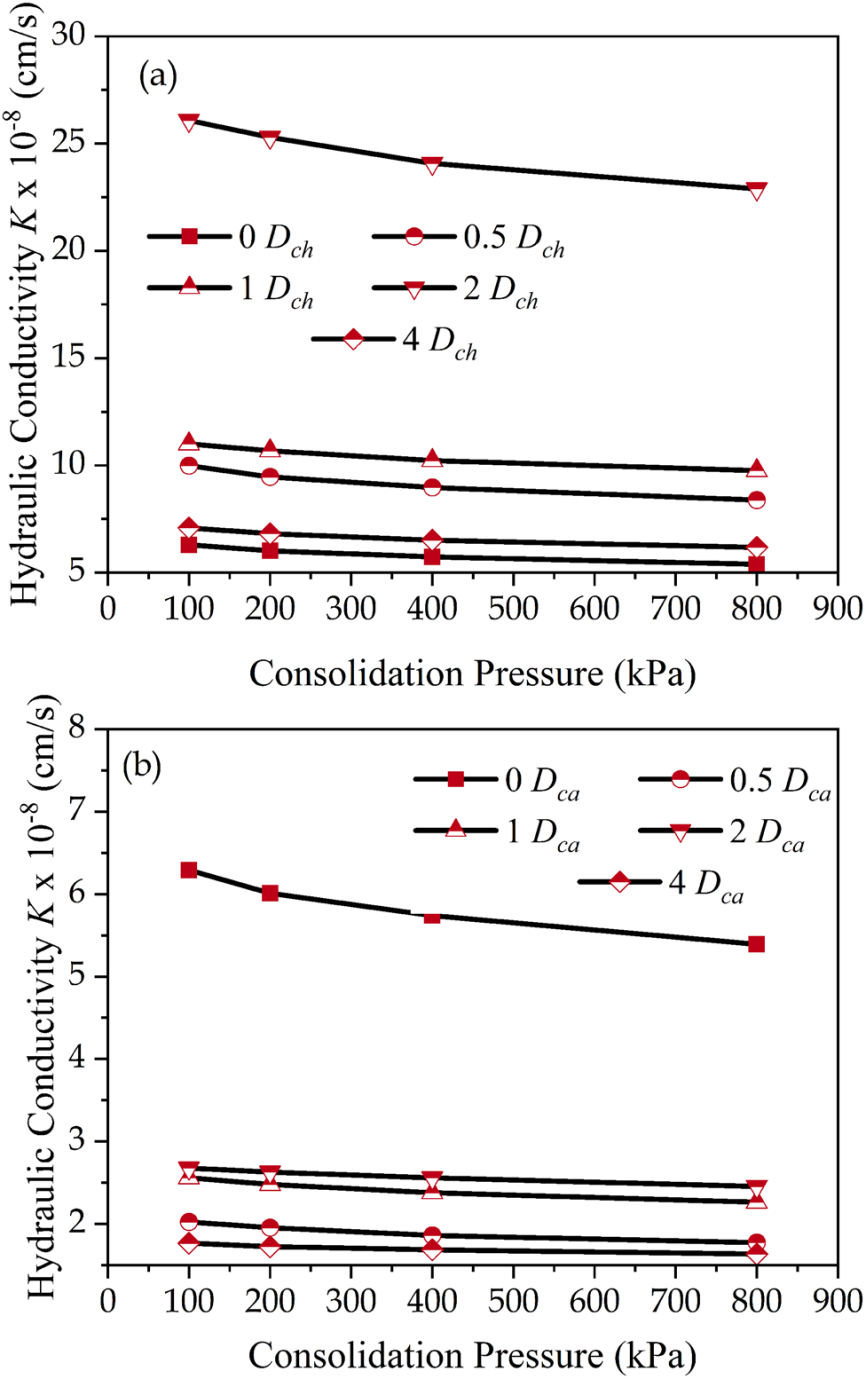
Variation of hydraulic conductivity (*K*) values (**a**) Chitosan-modified soil, (**b**) Casein-modified soil.

Figure 7a depicts the variation in hydraulic conductivity of soil-chitosan mixes for varying dosages (*D*_*ch*_) and consolidation pressures ($\sigma_{cp}$). The untreated soils attained *K* values of 6.29, 6.01, 5.74, and 5.39 (× 10^−8^ cm/s) indicating the lower permeability of the raw soil for consolidation pressures of 100, 200, 400, and 800 kPa. The *K* values declined as the consolidation pressure increased. After amending with chitosan (*D*_*ch*_), the *K* value increased by 1.57, 1.75, 4.15, and 1.13 times for dosages of 0.5%, 1%, 2%, and 4% at a consolidation pressure of 100 kPa. The hydraulic conductivity of soil treated with 4% (*D*_*ch*_) decreased by 0.27 times compared to chitosan amendment at 2%. However, at all dosages, the *K* values of soil-chitosan mixes were higher than those of raw soil. With a rise in consolidation pressure, the *K* values declined by 0.95, 0.89, and 0.84 times at *D*_*ch*_ = 0.5% at consolidation pressure of 200, 400, and 800 kPa. For *D*_*ch*_ > 0.5%, the *K* values decreased with an increase in consolidation pressure up to 800 kPa.

For the case of casein-treated soil, the hydraulic conductivity decreased by 0.32, 0.41, 0.43, and 0.28 times for *D*_*ca*_ = 0.5%, 1%, 2%, and 4% at $\sigma_{cp}$ = 100 kPa as shown in Fig. 7b. With a rise in consolidation pressure, the *K* values further dropped for all dosages (*D*_*ca*_). Considering the liner requirement as per *K* value, the casein-treated soils have exhibited a drop in *K* value at all dosages (*D*_*ca*_) whereas, the chitosan amendment brought a rise in *K* value at all dosages (*D*_*ch*_) compared to untreated soil. As displayed in Fig. 3, chitosan and casein amended samples took up an aggregated and flocculated structure. The interaction of chitosan and casein is due to the electrostatic force of attraction between them and clay particles. Unlike chemical stabilization, the soil-biopolymer bond does not contribute to the formation of any new compounds within the structure. For neutral biopolymers such as chitosan, the adsorption on the clay surface is largely entropy-driven and leads to the desorption of water molecules. The increase in entropy can be attributed to the breaking of sequential hydrogen bonding extending from the clay surface^51^. Anionic polymers or polyanions such as Casein are effective flocculants, especially while accompanying polyvalent cations such as exposed Aluminium ions at the clay surface^52^. The aforementioned interactions between chitosan and casein with clay particles led to the formation of a flocculated and aggregated structure. Chitosan acted as a voluminous filler material and increased the hydraulic conductivity with an increase in dosage. Whereas, casein formed a uniform coating over the soil particles and relatively led to the formation of a denser structure. This aided in reducing the value of hydraulic conductivity of soil-casein mixes.

### Effect of dosage on factors of safety ($FS_{{D_{ch} }}$ and $FS_{{D_{ca} }}$) of chitosan (***D***_***ch***_) and casein-treated (***D***_***ca***_) soils

**Figure 8:**
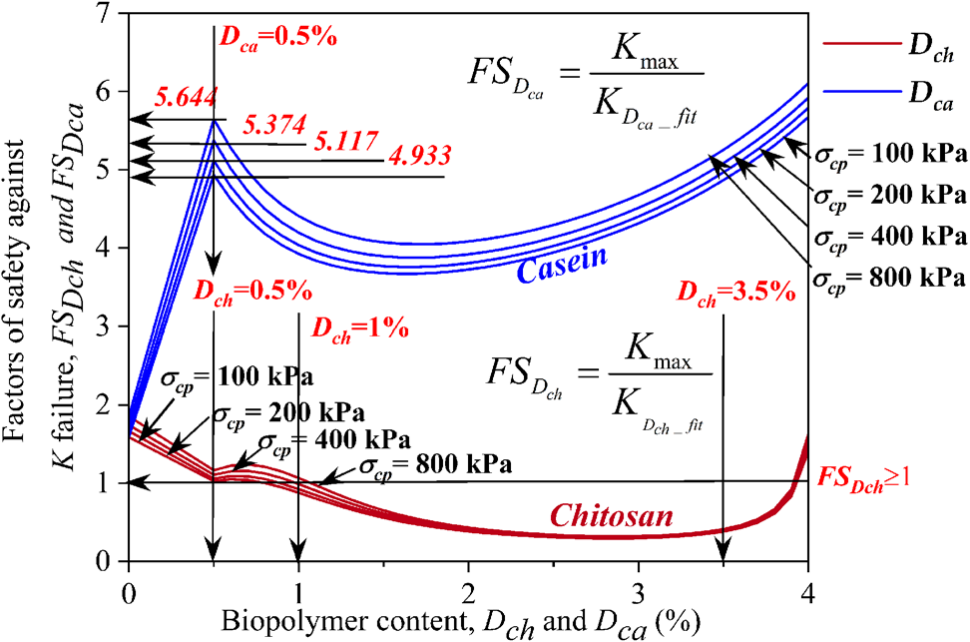
Effect of Chitosan (*D*_*ch*_) and Casein (*D*_*ca*_) contents on factors of safety of liner material when treated with Chitosan ($FS_{{D_{ch} }}$) and Casein ($FS_{{D_{ca} }}$) for different consolidation pressures ($\sigma_{cp}$).

The effect of dosage on the factors of safety modified using chitosan ($FS_{{D_{ch} }}$) and casein ($FS_{{D_{ca} }}$) is presented in Fig. 8. A factor of safety (*FS*) of 1.589 was attained for untreated soil at a consolidation pressure ($\sigma_{cp}$) of 100 kPa. As $\sigma_{cp}$ rose to 200, 400, and 800 kPa, the factor of safety increased by 4.78%, 9.75%, and 16.61% respectively. The increase in *FS* with an increase in $\sigma_{cp}$ can be attributed to the decrease in *K*_ch_ observed in Fig. 7a. On adding chitosan to the raw soil, the factors of safety against *K* failure decreased to 1.026, 1.058, 1.107, and 1.165 at $\sigma_{cp}$ = 100, 200, 400, and 800 kPa for dosage of 5% (*D*_*ch*_). The increase in hydraulic conductivity upon the addition of *D*_*ch*_ = 0.5% as shown in Fig. 7a can be a contributing factor to the decrease in $FS_{{D_{ch} }}$. The hydraulic conductivity values further increased at *D*_*ch*_ = 1% and this was reflected in the $FS_{{D_{ch} }}$ values attained for different $\sigma_{cp}$ at the same dosage. Beyond a dosage of 1%, the factors of safety decreased below 1 indicating the negative influence of higher dosages of chitosan.

However, $FS_{{D_{ch} }}$ started increasing beyond *D*_*ch*_ = 3.5% and attained values of $FS_{{D_{ch} }}$ = 1.412, 1.456, 1.534, and 1.633 at $\sigma_{cp}$ = 100, 200, 400, and 800 kPa respectively at *D*_*ch*_ = 4%. This is in agreement with the increase in hydraulic conductivity up to 2% and a decrease at 4% *D*_*ch*_ as shown in Fig. 7a. From Fig. 8, it can be observed that, unlike chitosan, casein has displayed a significant peak in $FS_{{D_{ca} }}$ after treating with dosage of 0.5% (Dca). This can be attributed to the decrease in hydraulic conductivity at the same dosage of casein as displayed in Fig. 7b. The rise in $FS_{{D_{ca} }}$ was increased by a factor of 3.05, 3.07, 3.08, and 3.05 times at $\sigma_{cp}$ = 100, 200, 400, and 800 kPa respectively. Beyond *D*_*ca*_ = 0.5%, a slight increase in hydraulic conductivity was observed (Fig. 7b) which led to a decrease in $FS_{{D_{ca} }}$ up to a dosage of 2%. Beyond *D*_*ca*_ = 2%, $FS_{{D_{ca} }}$ displayed a continuous increase up to the highest dosage of 4%. The highest $FS_{{D_{ca} }}$ of 8.272, 8.37, 8.348, and 8.643 were attained at *D*_*ca*_ = 4% for $\sigma_{cp}$ = 100, 200, 400, and 800 kPa respectively.

### Effect of dosage on reliability indices ($\beta_{Dch}$ and $\beta_{Dca}$) of chitosan (***D***_***ch***_) and casein-treated (***D***_***ca***_) soils

**Figure 9:**
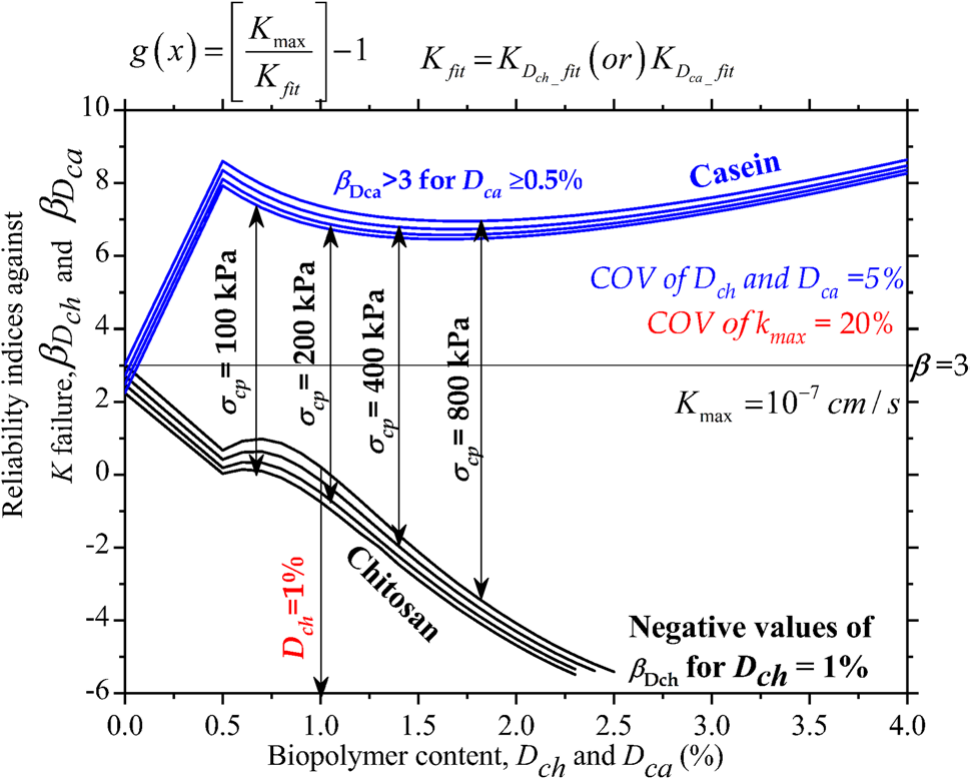
Effect of Chitosan (*D*_*ch*_) and Casein (*D*_*ca*_) contents on reliability indices of liner material when treated with Chitosan ($\beta_{Dch}$) and Casein ($\beta_{Dca}$) for different consolidation pressures ($\sigma_{cp}$).

The feasibility of utilizing the biopolymers, chitosan, and casein as a liner amendment for cohesive deposits was evaluated using the reliability indices. Figure 9 depicts the effect of biopolymer dosages on the reliability indices ($\beta_{Dch}$ and $\beta_{Dca}$). A reliability index of 3 is regarded as the optimum value to consider a material appropriate for the intended application. In the case of untreated soil, the material exhibited positive values ($\beta$) of 2.24, 2.475, 2.709, and 3.015 for $\sigma_{cp}$ = 100, 200, 400, and 800 kPa respectively. For untreated and biopolymer-treated soils, the samples exhibited an increase in reliability index with an increase in consolidation pressure. After amending the soil with *D*_*ch*_ = 0.5%, the reliability indices ($\beta_{Dch}$) reduced to 0.032, 0.187, 0.415, and 0.669 for an increase in $\sigma_{cp}$ from 100 to 800 kPa. The increase in permeability at *D*_*ch*_ = 0.5% as shown in Fig. 7a led to a dip in reliability indices. Further, the reliability indices dropped to negative values beyond *D*_*ch*_ = 0.5% indicating the detrimental effects of chitosan as a liner material.

For casein-treated soils, the reliability indices increased by 3.54, 3.28, 3.09, and 2.85 times after mixing soil with *D*_*ca*_ of 0.5% for $\sigma_{cp}$ = 100, 200, 400, and 800 kPa respectively. The reliability index achieved a value of greater than 3 for the different dosages of casein (*D*_*ca*_) admixed with soil. Even though the reliability indices ($\beta_{Dca}$) witnessed a drop at *D*_*ca*_ = 1% and 2%, the values further increased at *D*_*ca*_ = 4%. Casein proved to be an excellent liner material at all dosages by attaining $\beta_{Dca}$ > 3. The above reliability indices were compared for chitosan and casein-treated soils at COV of *K*_*max*_ = 20%.

### Comparison of reliability index of casein treated soil ($\beta_{Dca}$) for different ***COV of K***_***max***_ for different consolidation pressures

**Figure 10:**
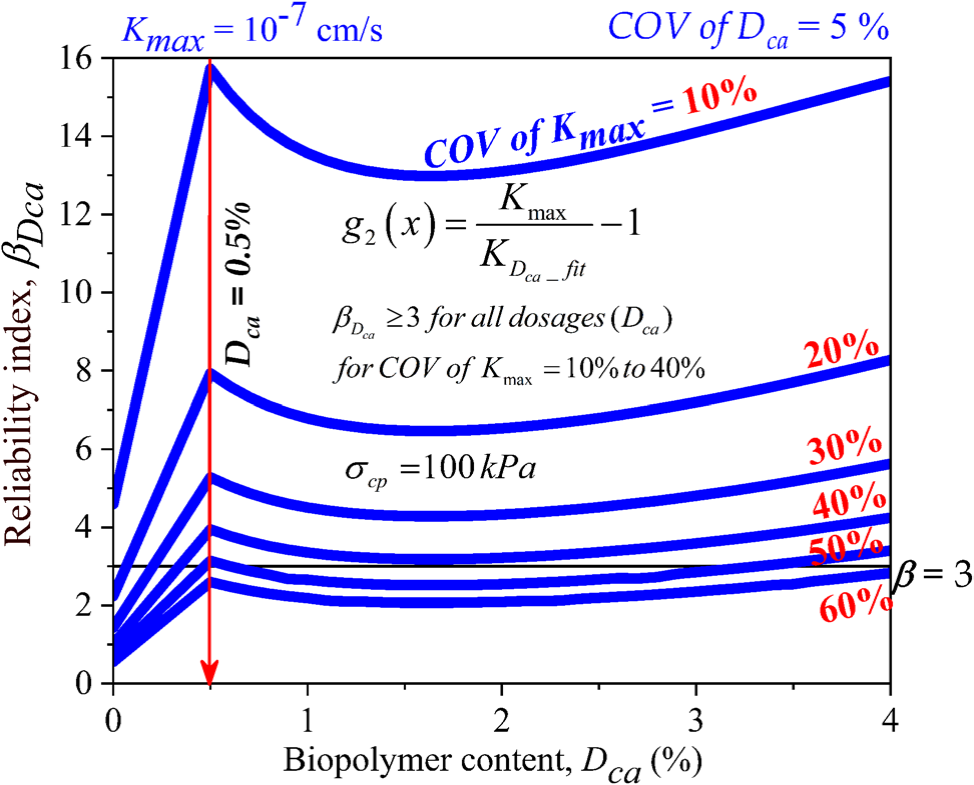
Effect of *COV* of *K*_*max*_ and dosages of Casein (*D*_*ca*_) on reliability index of liner material treated with Casein ($\beta_{Dca}$) with consolidation pressure ($\sigma_{cp}$) of 100 kPa.

After evaluating the reliability indices for chitosan and casein-treated soils for the different consolidation pressures, it was observed that casein completely outperformed chitosan as a liner material against *K* failure. Considering the performance of casein and reliability indices achieved at different dosages and consolidation pressures, the casein-treated soils were only considered for assessing the effect of *COV of K*_*max*_. The effect of *COV of K*_*max*_ on the reliability indices of casein-treated soils for all dosages at a consolidation pressure of 100 kPa is shown in Fig. 10. The untreated soil exhibited reliability indices of 4.595, 2.24, 1.431, 1.01, 0.745, and 0.558 at *COV of K*_*max*_ = 10%, 20%, 30%, 40%, 50%, and 60% respectively. As the *COV of K*_*max*_ increased, the reliability indices dropped indicating detrimental effects due to the uncertainties involved within the environmental conditions. The general trend for reliability index was a steep increase at *D*_*ca*_ = 0.5%, followed by a drop at 1% and 2% and further an increase in reliability index up to the highest dosage of 4%. At *D*_*ca*_ = 0.5%, a reliability index ($\beta_{Dca}$) of 3 was attained for *COV of K*_*max*_ upto 50%. At *COV of K*_*max*_ = 60%, the highest value achieved is 2.83 for a dosage of 4%.

**Figure 11:**
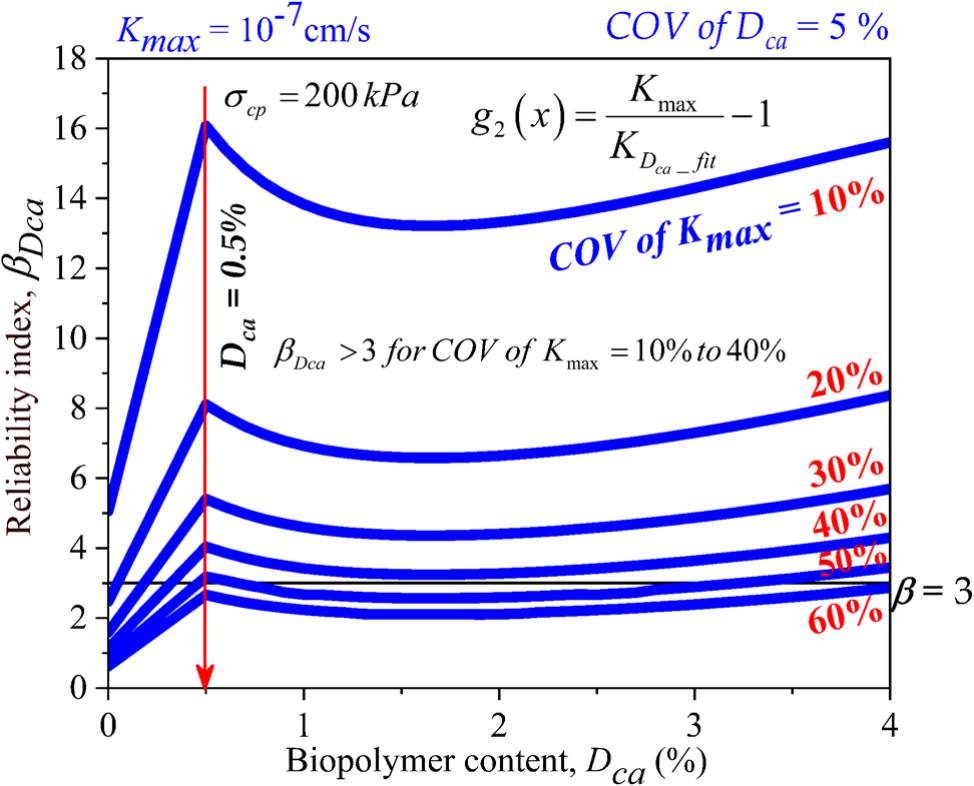
Effect of *COV* of *K*_*max*_ and dosages of Casein (*D*_*ca*_) on reliability index of liner material treated with Casein ($\beta_{Dca}$) with consolidation pressure ($\sigma_{cp}$) of 200 kPa.

As the consolidation pressure was increased to 200 kPa, the trend remains the same. However, the reliability indices were observed to attain a rise at each dosage as shown in Fig. 11, compared to the values obtained at 100 kPa. This can be attributed to the decrease in *K* value with increase in consolidation pressure for the same dosage as shown in Fig. 7b. The peak values of reliability indices were attained as 16.076, 8.111, 5.404, 4.04, 3.185, and 2.665 at *D*_*ca*_ = 0.5% at *COV of K*_*max*_ = 10, 20, 30, 40, 50, and 60% respectively. Similar to the observation made for Fig. 10, a reliability index of 3 could not be achieved when the *COV of K*_*max*_ exceeded 50%.

**Figure 12:**
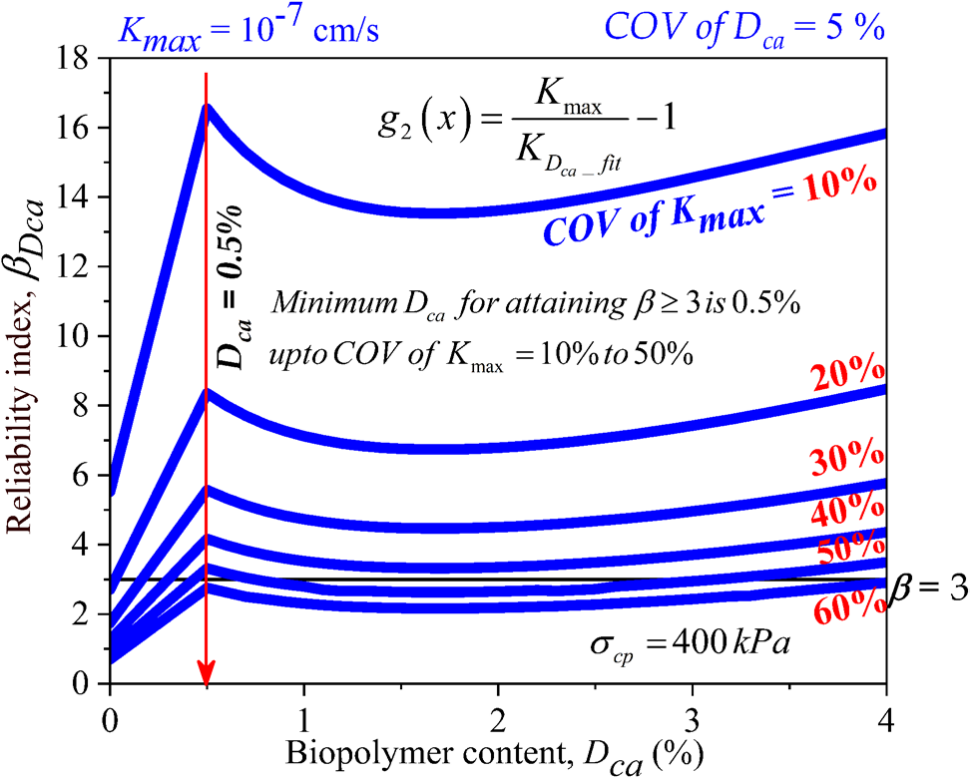
Effect of *COV* of *K*_*max*_ and dosages of Casein (*D*_*ca*_) on reliability index of liner material treated with Casein ($\beta_{Dca}$) with consolidation pressure ($\sigma_{cp}$) of 400 kPa.

**Figure 13:**
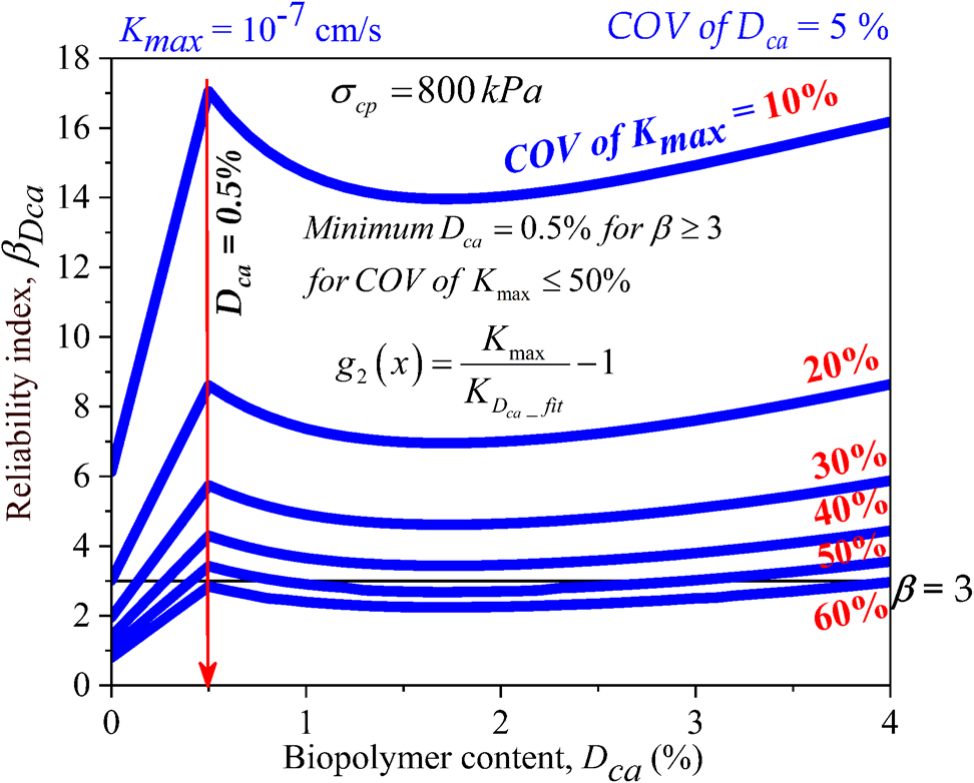
Effect of *COV* of *K*_*max*_ and dosages of Casein (*D*_*ca*_) on reliability index of liner material treated with Casein ($\beta_{Dca}$) with consolidation pressure ($\sigma_{cp}$) of 800 kPa.

At consolidation pressure of 400 kPa and 800 kPa, $\beta_{Dca}$ > 3 was attained at *D*_*ca*_ = 0.5% for *COV of K*_*max*_ up to 50% as displayed in Figs. 12 and 13. From Figs. 10, 11, 12 and 13, the importance of assessing the stability of the mixes under different uncertainties quantified in the form of *COV* on the hydraulic conductivity was established. Different factors such as compaction process, change in moisture content, influence of leachate concentrations, presence of organic and inorganic contaminants, can be considered as the varying factors affecting the liner integrity. These varying factors were quantified using *COV of K*_*max*_ in the current study. From analyzing the effect of *COV of K*_*max*_ on the reliability indices of casein-treated soils, it has been understood that the target reliability index of 3 can be achieved at the minimum dosage (*D*_*ca*_) of 0.5% and up to *COV of K*_*max*_ value of 50%. Thus, *D*_*ca*_ = 0.5% can be considered as the optimum dosage requirement for casein as a liner material amendment in soft soil.

## Conclusions

The influence of two hydrophobic biopolymers, namely chitosan and casein on the consolidation parameters of an organic clay has been investigated in the current study. Non-linear multivariate regression models were developed to predict the coefficient of consolidation (*C*_*v*_), hydraulic conductivity (*K*), primary compression index (*C*_*c*_), and secondary compression index (*C*_*α*_) values. To assess the feasibility of the biopolymer as a liner material, the probability of the liner against hydraulic conductivity failure was evaluated by conducting a reliability analysis. The following conclusions are drawn from the current study:

- The interaction between chitosan and casein with the soil particles led to the formation of an aggregated and flocculated structure and reduced the soil volume. At the highest consolidation pressure of 800 kPa, the primary compressibility (*C*_*c*_) was reduced by 18% and 59% at a maximum dosage (*D*_*ch*_ and *D*_*ca*_) of 4%.
- The secondary compression index (*C*_*α*_) was reduced by 32% and 47% for chitosan and casein-amended samples at 4% (*D*_*ch*_ and *D*_*ca*_) at a consolidation pressure of 800 kPa. The compressibility indices (*C*_*c*_ and *C*_*α*_) increased with a rise in consolidation pressure due to the disruption of the stable structures.
- The hydrophobic biopolymers also accelerated the consolidation process and increased the coefficient of consolidation (*C*_*v*_) at all dosages (*D*_*ch*_) and 2% *D*_*ca*_. The *C*_*v*_ values were found to reduce with a rise in consolidation pressure at all dosages (*D*_*ch*_ and *D*_*ca*_).
- In the case of chitosan-amended samples, the hydraulic conductivity increased compared to untreated soil whereas the casein-amended samples exhibited hydraulic conductivities lower than those of untreated soil. Thus, casein exhibited a potential application as a liner material.
- The addition of chitosan rendered factors of safety below 1 between dosages (*D*_*ch*_) of 0.5% and 3.5%, beyond which factors of safety attained values greater than 1 at all consolidation pressures. However, casein-amended samples exhibited factors of safety greater than 1 at all dosages and consolidation pressures.
- Chitosan amended liner material failed to achieve the target reliability index of 3 at all dosages. Whereas, the casein amended samples attained the target reliability index of 3 at all dosages and consolidation pressures at *COV of K*_*max*_ = 20%.
- At all consolidation pressures, the highest reliability indices (> 3) were attained at a dosage of 0.5% (*D*_*ca*_) for the different *COV of K*_*max*_ from 10 to 60%. Thus, the optimum dosage of casein required for liner amendment can be considered as *D*_*ca*_ = 0.5%.

Among the selected biopolymers for the current study, casein proved to be an excellent material for amending liner materials and exhibited higher factors of safety (> 1) and reliability indices (> 3) for all dosages (*D*_*ca*_). Biopolymers have proven to be environment friendly and non-polluting by virtue of the natural materials used for their synthesis. Either dry mixing method or wet mixing method can be adopted for achieving a homogeneous soil-biopolymer mix. As a future scope of work, the interaction of casein with various contaminants can be explored.

### Electronic supplementary material

Below is the link to the electronic supplementary material.

Supplementary Tables.

## Data Availability

All data generated or analysed during this study are included in this published article [and its supplementary information files].

## Appendix

### Primary compression index (C_c_) of untreated and biopolymer modified organic clay

Equation (4) presents the multivariate regression model for predicting Primary Compression index (*C*_*c*_) of untreated soil with R^2^ = 0.99 and $R_{adj}^{2}$ = 0.99.

4 $$C_{c\_ut\_fit} = {0}{\text{.2707}} + {3}{\text{.7933}} \times {10}^{{ - 9}} \times P^{2.5} + {0}{\text{.0028}} \times \log \left( P \right)$$

Equation (5) presents the multivariate regression model for predicting Primary Compression index (*C*_*c*_) of chitosan-treated soil (*D*_*ch*_) with R^2^ = 0.99 and $R_{adj}^{2}$ = 0.98.

5 $$C_{c\_Ch\_fit} \, = \left( \begin{gathered} {0}{\text{.4306}} - \frac{{{32}{\text{.30}}}}{P} - \frac{{{0}{\text{.5376}}}}{{D_{Ch} }}{ + }\frac{{{534}{\text{.55}}}}{{P^{2} }}{ + }\frac{{{0}{\text{.4850}}}}{{D_{Ch}^{{2}} }}{ + }\frac{{{22}{\text{.09}}}}{{P \times D_{Ch} }} \hfill \\ \;\;{ + }\frac{{{44190}{\text{.48}}}}{{P^{{3}} }} - \frac{{{0}{\text{.1112}}}}{{D_{Ch}^{{3}} }} - \frac{{{4}{\text{.48}}}}{{P \times D_{Ch}^{{2}} }} - \frac{{{1181}{\text{.74}}}}{{P^{{2}} \times D_{Ch} }} \hfill \\ \end{gathered} \right)$$

Equation (6) presents the multivariate regression model for predicting Primary Compression index (*C*_*c*_) of casein-treated soil (*D*_*ca*_) with R^2^ = 0.99 and $R_{adj}^{2}$ = 0.96.

6 $$C_{c\_Ca\_fit} \, = \left( \begin{gathered} - {0}{\text{.1538 + 0}}{.0024} \times P{ + 0}{\text{.0096}} \times {\text{log}}\left( {D_{Ca} } \right) - {4}{\text{.933}} \times 10^{ - 6} \times P^{{2}} - {0}{\text{.0115}} \times {\text{log}}\left( {D_{Ca} } \right)^{{2}} \hfill \\ \;\; - {0}{\text{.0005}} \times P \times {\text{log}}\left( {D_{Ca} } \right){ + 3}{\text{.2133}} \times 10^{ - 9} \times P^{{3}} { + 0}{\text{.0451}} \times {\text{log}}\left( {D_{Ca} } \right)^{{3}} \hfill \\ \;\; - {0}{\text{.0001}} \times P \times {\text{log}}\left( {D_{Ca} } \right)^{{2}} { + 5}{\text{.3724}} \times 10^{ - 7} \times P^{{2}} \times {\text{log}}\left( {D_{Ca} } \right) \hfill \\ \end{gathered} \right)$$

### Secondary compression index (C_α_) of untreated and biopolymer modified organic clay

Equation (7) presents the multivariate regression model for predicting Secondary Compression index (*C*_*α*_) of untreated soil with R^2^ = 0.99 and $R_{adj}^{2}$ = 0.99.

7 $$C_{\alpha \_ut\_fit} = {0}{\text{.02433}} - {6}{\text{.2507}} \times {10}^{{ - 8}} \times P^{1.5} - 0.265 \times \frac{\log \left( P \right)}{P}$$

Equation (8) presents the multivariate regression model for predicting Secondary Compression index (*C*_*α*_) of chitosan-treated soil (*D*_*ch*_) with R^2^ = 0.89 and $R_{adj}^{2}$ = 0.72.

8 $$C_{\alpha \_Ch\_fit} \, = \, \left( \begin{gathered} {0}{\text{.02066 + 2}}{.0811} \times {10}^{{ - 6}} \times P - {0}{\text{.02160}} \times D_{Ch} - {7}{\text{.2493}} \times {10}^{{ - 9}} \times P^{{2}} { + 0}{\text{.01072}} \times D_{Ch}^{{2}} \hfill \\ \;\;{ + 1}{\text{.2348}} \times {10}^{ - 5} \times P \times D_{Ch} { + 8}{\text{.8839}} \times {10}^{{ - 12}} \times P^{{3}} - 0.00{1489} \times D_{Ch}^{{3}} \hfill \\ \;\; - {3}{\text{.2335}} \times {10}^{{ - 6}} \times P \times D_{Ch}^{{2}} { + 1}{\text{.6830}} \times {10}^{{ - 9}} \times P^{{2}} \times D_{Ch} \hfill \\ \end{gathered} \right)$$

Equation (9) presents the multivariate regression model for predicting Secondary Compression index (*C*_*α*_) of casein-treated soil (*D*_*ca*_) with R^2^ = 0.99 and $R_{adj}^{2}$ = 0.99.

9 $$C_{\alpha \_Ca\_fit} \, = \left( \begin{gathered} {0}{\text{.0202}} - \frac{{{1}{\text{.9054}}}}{P} - \frac{{{0}{\text{.0081}}}}{{D_{Ca} }}{ + }\frac{{{158}{\text{.10}}}}{{P^{2} }}{ + }\frac{{{0}{\text{.0022}}}}{{D_{Ca}^{{2}} }}{ + 1}{\text{.0078}} \times \frac{{D_{Ca} }}{P} \hfill \\ \;\; - \frac{{{11809}{\text{.5237}}}}{{P^{{3}} }} - \frac{{{0}{\text{.0002}}}}{{D_{Ca}^{{3}} }} - {0}{\text{.1210}}\frac{{D_{Ca}^{2} }}{P} - {34}{\text{.90}}\frac{{D_{Ca} }}{{P^{2} }} \hfill \\ \end{gathered} \right)$$

### Coefficient of consolidation (C_v_) of untreated and biopolymer modified organic clay

Equation (10) presents the multivariate regression model for predicting Coefficient of Consolidation (*C*_*v*_) of untreated soil with R^2^ = 0.99 and $R_{adj}^{2}$ = 0.99.

10 $$C_{v\_ut\_fit} = 6.4301 - 0.0385 \times P^{5} + \frac{190.1531}{{P^{1.5} }}$$

Equation (11) presents the multivariate regression model for predicting Coefficient of Consolidation (*C*_*v*_) of chitosan-treated soil with R^2 ^= 0.99 and $R_{adj}^{2}$ = 0.99.

11 $$C_{v\_Ch\_fit} \, = \left( \begin{gathered} - {16}{\text{.3743 + 10}}{.4219} \times {\text{log}}\left( P \right){ + 12}{\text{.0708}} \times D_{Ch} - {1}{\text{.6304}} \times {\text{log}}\left( P \right)^{{2}} \hfill \\ \;\;\;{ + 15}{\text{.7542}} \times D_{Ch}^{{2}} - {2}{\text{.5890}} \times {\text{log}}\left( P \right) \times D_{Ch} { + 0}{\text{.0894}} \times {\text{log}}\left( P \right)^{{3}} \hfill \\ \;\; - {4}{\text{.5380}} \times D_{Ch}^{{3}} { + 0}{\text{.5911}} \times {\text{log}}\left( P \right) \times D_{Ch}^{{2}} - {0}{\text{.0098}} \times {\text{log}}\left( P \right)^{{2}} \times D_{Ch} \hfill \\ \end{gathered} \right)$$

Equation (12) presents the multivariate regression model for predicting Coefficient of Consolidation (*C*_*v*_) of casein-treated soil with R^2^ = 0.99 and $R_{adj}^{2}$ = 0.99.

12 $$C_{v\_Ca\_fit} \, = \left( \begin{gathered} {2}{\text{.5974}} - {0}{\text{.0019}} \times P{ + 2}{\text{.0858}} \times D_{Ca} { + 2}{\text{.8283}} \times 10^{ - 6} \times P^{{2}} { + 0}{\text{.6114}} \times D_{Ca}^{{2}} \hfill \\ \;\; - {0}{\text{.0003}} \times P \times D_{Ca} - {1}{\text{.5415}} \times 10^{ - 9} \times P^{{3}} - {0}{\text{.2457}} \times D_{Ca}^{{3}} \hfill \\ \;\;{ + 0}{\text{.0001}} \times P \times D_{Ca}^{{2}} - {8}{\text{.0508}} \times 10^{ - 8} \times P^{{2}} \times D_{Ca} \hfill \\ \end{gathered} \right)$$

### Hydraulic conductivity (K) of untreated and biopolymer modified organic clay

Equation (13) presents the multivariate regression model for predicting Hydraulic conductivity (*K*) of untreated soil with R^2^ = 0.99 and $R_{adj}^{2}$ = 0.99.

13 $$K_{ut\_fit} = \left( {{6}{\text{.4876 - 0}}{.03885} \times \sqrt P + \frac{192.85}{{P^{1.5} }}} \right) \times \,10^{ - 8}$$

Equation (14) presents the multivariate regression model for predicting Hydraulic conductivity (*K*) of chitosan-treated soil with R^2^ = 0.99 and $R_{adj}^{2}$ = 0.99.

14 $$K_{Ch\_fit} \, = \, \left( \begin{gathered} {5}{\text{.8799}} + {5}{\text{.2408}} \times \log \left( P \right) - {17}{\text{.8283}} \times D_{Ch} - {0}{\text{.8620}} \times \log \left( P \right)^{2} \hfill \\ \;\; + {21}{\text{.6984}} \times D_{Ch}^{2} - {1}{\text{.1963}} \times \log \left( P \right) \times D_{Ch} + 0.0{4643} \times \log \left( P \right)^{3} \hfill \\ \;\; - {4}{\text{.4304}} \times D_{Ch}^{3} + 0.{2844} \times \log \left( P \right) \times D_{Ch}^{2} - {0}{\text{.0049}} \times \log \left( P \right)^{2} \times D_{Ch} \hfill \\ \end{gathered} \right) \times 10^{ - 8}$$

Equation (15) presents the multivariate regression model for predicting Hydraulic conductivity (*K*) of casein-treated soil with R^2^ = 0.99 and $R_{adj}^{2}$ = 0.99.

15 $$K_{Ca\_fit} \, = \, \left( \begin{gathered} {2}{\text{.6402}} - {0}{\text{.001}} \times P{ + }0.{6239} \times {\text{log}}\left( {D_{Ca} } \right){ + 1}{\text{.2293}} \times P^{2} - {0}{\text{.4112}} \times {\text{log}}\left( {D_{Ca} } \right)^{2} \hfill \\ \;\;{ + }0.000{15} \times P \times {\text{log}}\left( {D_{Ca} } \right) - {6}{\text{.5944}} \times P^{3} - {0}{\text{.3374}} \times {\text{log}}\left( {D_{Ca} } \right)^{3} \hfill \\ \;\;{ + }0.000{1} \times P \times {\text{log}}\left( {D_{Ca} } \right)^{2} - {1}{\text{.4603}} \times P^{2} \times {\text{log}}\left( {D_{Ca} } \right) \hfill \\ \end{gathered} \right) \times 10^{ - 8}$$

## List of symbols

*COV*: Coefficient of variation
*FS*: Factors of safety against hydraulic conductivity failure of untreated soil
$FS_{{D_{ch} }}$: Factors of safety against hydraulic conductivity failure of Chitosan treated soil
$FS_{{D_{ca} }}$: Factors of safety against hydraulic conductivity failure of Casein treated soil
*D*_*ch*_: Chitosan content
*g*_*1*_(*x*): The limit state function for Chitosan-treated soil
*D*_*ca*_: Casein content
*g*_*2*_(*x*): The limit state function for Casein-treated soil
*K*: Hydraulic conductivity of soil
$K_{ut\_\exp }$: Hydraulic conductivity of untreated soil obtained from the experiment
$K_{ut\_fit}$: Hydraulic conductivity of untreated soil obtained from curve fitting
$K_{Chi\_\exp }$: Hydraulic conductivity of *D*_*ch*_ modified soil obtained from experiment
$K_{Chi\_fit}$: Hydraulic conductivity of *D*_*ch*_ modified soil obtained from curve fitting
$K_{Ca\_\exp }$: Hydraulic conductivity of *D*_*ca*_ modified soil obtained from experiment
$K_{Ca\_fit}$: Hydraulic conductivity of *D*_*ca*_ modified soil obtained from curve fitting
*K*_*max*_: Maximum specified value of hydraulic conductivity as per the regulatory standards
$C_{v\_ut\_\exp }$: Coefficient of consolidation of untreated soil obtained from experiment
$C_{v\_ut\_fit}$: Coefficient of consolidation of untreated soil obtained from curve fitting
$C_{v\_Chi\_\exp }$: Coefficient of consolidation of *D*_*ch*_ modified soil obtained from experiment
$C_{v\_Chi\_fit}$: Coefficient of consolidation of *D*_*ch*_ modified soil obtained from curve fitting
$C_{v\_Ca\_\exp }$: Coefficient of consolidation of *D*_*ca*_ modified soil obtained from experiment
$C_{v\_Ca\_fit}$: Coefficient of consolidation of *D*_*ca*_ modified soil obtained from curve fitting
$C_{c\_ut\_\exp }$: Primary compression index of untreated soil obtained from experiment
$C_{c\_ut\_fit}$: Primary compression index of untreated soil obtained from curve fitting
$C_{c\_Chi\_\exp }$: Primary compression index of *D*_*ch*_ modified soil obtained from experiment
$C_{c\_Chi\_fit}$: Primary compression index of *D*_*ch*_ modified soil obtained from curve fitting
$C_{c\_Ca\_\exp }$: Primary compression index of *D*_*ca*_ modified soil obtained from experiment
$C_{c\_Ca\_fit}$: Primary compression index of *D*_*ca*_ modified soil obtained from curve fitting
$C_{\alpha \_ut\_\exp }$: Secondary compression index of untreated soil obtained from experiment
$C_{\alpha \_ut\_fit}$: Secondary compression index of untreated soil obtained from curve fitting
$C_{\alpha \_Chi\_\exp }$: Secondary compression index of *D*_*ch*_ modified soil obtained from experiment
$C_{\alpha \_Chi\_fit}$: Secondary compression index of *D*_*ch*_ modified soil obtained from curve fitting
$C_{\alpha \_Ca\_\exp }$: Secondary compression index of *D*_*ca*_ modified soil obtained from experiment
$C_{\alpha \_Ca\_fit}$: Secondary compression index of *D*_*ca*_ modified soil obtained from curve fitting
$$\beta$$: Reliability index
$\beta_{Dch}$: Reliability index against hydraulic conductivity failure of Chitosan treated soil
$\beta_{Dca}$: Reliability index against hydraulic conductivity failure of Casein treated soil
$$\sigma_{cp}$$, *P*: Consolidation pressure
