# Supplementary material for: Target reliability-based design optimization studies on cohesive soil amended with chitosan and casein for liner applications
Source: Sci Rep. 2024 Jun 20;14:14202. doi: 10.1038/s41598-024-64408-7 (PMC11190237; doi:10.1038/s41598-024-64408-7)
Supplement: Supplementary file 1 — Supplementary Tables. [file 41598_2024_64408_MOESM1_ESM.docx]

**Supplementary Material**

**Table S1. The comparison of experimental values of coefficient of permeability (**$K_{ut\_\exp}$**) and best-fit coefficient of permeability (**$K_{ut\_fit}$**) of untreated clay using consolidation pressure (**$P$**) of 100, 200, 400, and 800 kPa**

| $P$  (kPa) | $K_{ut\_\exp}$ $\times10^{-8}$  (cm/s) | $K_{ut\_fit}$ $\times10^{-8}$   (cm/s) | Residual $\times10^{-12}$  (cm/s) | % error |
| --- | --- | --- | --- | --- |
| 100 | 6.292 | 6.2919 | 0.6880 | 0.001094 |
| 200 | 6.006 | 6.0063 | -3.1227 | -0.005199 |
| 400 | 5.735 | 5.7346 | 3.8112 | 0.006646 |
| 800 | 5.397 | 5.3971 | -1.3770 | -0.002551 |

**Table S2. The comparison of experimental values of coefficient of permeability (**$K_{Chi\_\exp}$**) and best-fit coefficient of permeability (**$K_{Chi\_fit}$**) of treated clay with Chitosan using consolidation pressure (**$P$**) of 100, 200, 400, and 800 kPa**

| $P$  (kPa) | $D_{Ch}$  (%) | $K_{Chi\_\exp}$ $\times10^{-8}$  (cm/s) | $K_{Chi\_fit}$ $\times10^{-8}$   (cm/s) | Residual $\times10^{-8}$  (cm/s) | % error |
| --- | --- | --- | --- | --- | --- |
| 100 | 0.5 | 9.9898 | 9.7446 | 0.2452 | 2.45 |
|  | 1.0 | 11.049 | 11.403 | -0.354 | -3.20 |
|  | 2.0 | 26.080 | 25.972 | 0.1076 | 0.41 |
|  | 4.0 | 7.0833 | 7.0821 | 0.0012 | 0.02 |
| 200 | 0.5 | 9.4613 | 9.4486 | 0.0126 | 0.13 |
|  | 1.0 | 10.681 | 10.823 | -0.1422 | -1.33 |
|  | 2.0 | 25.295 | 25.121 | 0.174 | 0.69 |
|  | 4.0 | 6.8260 | 6.8705 | -0.0445 | -0.65 |
| 400 | 0.5 | 8.9740 | 9.0310 | -0.057 | -0.64 |
|  | 1.0 | 10.234 | 10.120 | 0.1145 | 1.12 |
|  | 2.0 | 24.086 | 24.141 | -0.0548 | -0.23 |
|  | 4.0 | 6.5180 | 6.5206 | -0.0026 | -0.04 |
| 800 | 0.5 | 8.3838 | 8.5846 | -0.2008 | -2.40 |
|  | 1.0 | 9.7663 | 9.3846 | 0.3817 | 3.91 |
|  | 2.0 | 22.898 | 23.125 | -0.2267 | -0.99 |
|  | 4.0 | 6.1712 | 6.1253 | 0.04591 | 0.74 |

**Table S3. The comparison of experimental values of coefficient of permeability (**$K_{Ca\_\exp}$**) and best-fit coefficient of permeability (**$K_{Ca\_fit}$**) of treated clay with Casein using consolidation pressure (**$P$**) of 100, 200, 400, and 800 kPa**

| $P$  (kPa) | $D_{Ca}$  (%) | $K_{Ca\_\exp}$ $\times10^{-8}$  (cm/s) | $K_{Ca\_fit}$ $\times10^{-8}$   (cm/s) | Residual $\times10^{-8}$  (cm/s) | % error |
| --- | --- | --- | --- | --- | --- |
| 100 | 0.5 | 2.023 | 2.027 | -0.00446 | -0.22 |
|  | 1.0 | 2.559 | 2.549 | 0.009637 | 0.38 |
|  | 2.0 | 2.677 | 2.686 | -0.00947 | -0.35 |
|  | 4.0 | 1.768 | 1.763 | 0.004289 | 0.24 |
| 200 | 0.5 | 1.957 | 1.954 | 0.002549 | 0.13 |
|  | 1.0 | 2.479 | 2.479 | 0.00 | 0.00 |
|  | 2.0 | 2.627 | 2.628 | -0.0015 | -0.06 |
|  | 4.0 | 1.726 | 1.727 | -0.00109 | -0.06 |
| 400 | 0.5 | 1.861 | 1.861 | -0.00016 | -0.01 |
|  | 1.0 | 2.380 | 2.385 | -0.00454 | -0.19 |
|  | 2.0 | 2.559 | 2.552 | 0.006423 | 0.25 |
|  | 4.0 | 1.688 | 1.690 | -0.00173 | -0.10 |
| 800 | 0.5 | 1.774 | 1.772 | 0.002066 | 0.12 |
|  | 1.0 | 2.264 | 2.269 | -0.00515 | -0.23 |
|  | 2.0 | 2.454 | 2.450 | 0.00455 | 0.19 |
|  | 4.0 | 1.637 | 1.639 | -0.00147 | -0.09 |

**Table S4. The comparison of experimental values of coefficient of consolidation (****) and best-fit coefficient of consolidation (****) of untreated clay using consolidation pressure (**$P$**) of 100, 200, 400, and 800 kPa**

| $P$  (kPa) |   (m^2^/year) |   (m^2^/year) | Residual  (m^2^/year) | % error |
| --- | --- | --- | --- | --- |
| 100 | 6.2351 | 6.2350 | 0.00005 | 0.0009 |
| 200 | 5.9523 | 5.9525 | -0.00024 | -0.0040 |
| 400 | 5.6837 | 5.6834 | 0.00029 | 0.0052 |
| 800 | 5.3488 | 5.3489 | -0.00011 | -0.0020 |

**Table S5. The comparison of experimental values of coefficient of consolidation (****) and best-fit coefficient of consolidation (****) of treated clay with Chitosan using consolidation pressure (**$P$**) of 100, 200, 400, and 800 kPa**

| $P$  (kPa) | $D_{Ch}$  (%) |   (m^2^/year) |  (m^2^/year) | Residual  (m^2^/year) | % error |
| --- | --- | --- | --- | --- | --- |
| 100 | 0.5 | 10.080 | 9.795 | 0.285 | 2.83 |
|  | 1.0 | 19.278 | 19.652 | -0.374 | -1.94 |
|  | 2.0 | 43.323 | 43.255 | 0.068 | 0.16 |
|  | 4.0 | 10.744 | 10.723 | 0.021 | 0.19 |
| 200 | 0.5 | 9.546 | 9.563 | -0.016 | -0.17 |
|  | 1.0 | 18.635 | 18.796 | -0.161 | -0.87 |
|  | 2.0 | 42.019 | 41.767 | 0.252 | 0.60 |
|  | 4.0 | 10.354 | 10.428 | -0.074 | -0.72 |
| 400 | 0.5 | 9.055 | 9.124 | -0.070 | -0.77 |
|  | 1.0 | 17.855 | 17.729 | 0.126 | 0.71 |
|  | 2.0 | 40.010 | 40.058 | -0.049 | -0.12 |
|  | 4.0 | 9.887 | 9.894 | -0.008 | -0.08 |
| 800 | 0.5 | 8.459 | 8.658 | -0.199 | -2.35 |
|  | 1.0 | 17.039 | 16.630 | 0.409 | 2.40 |
|  | 2.0 | 38.037 | 38.308 | -0.271 | -0.71 |
|  | 4.0 | 9.361 | 9.299 | 0.061 | 0.65 |

**Table S6. The comparison of experimental values of coefficient of consolidation (****) and best-fit coefficient of consolidation (****) of treated clay with Casein using consolidation pressure (**$P$**) of 100, 200, 400, and 800 kPa**

| $P$  (kPa) | $D_{Ca}$  (%) |   (m^2^/year) |  (m^2^/year) | Residual  (m^2^/year) | % error |
| --- | --- | --- | --- | --- | --- |
| 100 | 0.5 | 3.576 | 3.585 | -0.009 | -0.26 |
|  | 1.0 | 4.878 | 4.863 | 0.015 | 0.30 |
|  | 2.0 | 7.048 | 7.061 | -0.013 | -0.19 |
|  | 4.0 | 4.867 | 4.859 | 0.008 | 0.16 |
| 200 | 0.5 | 3.460 | 3.454 | 0.005 | 0.16 |
|  | 1.0 | 4.726 | 4.724 | 0.002 | 0.05 |
|  | 2.0 | 6.916 | 6.917 | -0.001 | -0.01 |
|  | 4.0 | 4.753 | 4.760 | -0.007 | -0.15 |
| 400 | 0.5 | 3.290 | 3.295 | -0.005 | -0.16 |
|  | 1.0 | 4.538 | 4.544 | -0.007 | -0.15 |
|  | 2.0 | 6.737 | 6.724 | 0.013 | 0.19 |
|  | 4.0 | 4.647 | 4.648 | -0.001 | -0.02 |
| 800 | 0.5 | 3.136 | 3.127 | 0.009 | 0.28 |
|  | 1.0 | 4.317 | 4.327 | -0.011 | -0.24 |
|  | 2.0 | 6.462 | 6.461 | 0.001 | 0.02 |
|  | 4.0 | 4.507 | 4.507 | 0.001 | 0.01 |

**Table S7. The comparison of experimental values of primary compression index (****) and best-fit primary compression index (****) of untreated clay using consolidation pressure (**$P$**) of 100, 200, 400, and 800 kPa**

| $P$  (kPa) |  |  | Residual | % error |
| --- | --- | --- | --- | --- |
| 100 | 0.2840 | 0.2840 | 5.01$\times10^{-6}$ | 0.0018 |
| 200 | 0.2877 | 0.2877 | -10.9$\times10^{-6}$ | -0.0038 |
| 400 | 0.2996 | 0.2996 | 6.8$\times10^{-6}$ | 0.0023 |
| 800 | 0.3581 | 0.3581 | -0.89$\times10^{-6}$ | -0.0002 |

**Table S8. The comparison of experimental values of primary compression index (****) and best-fit primary compression index (****) of treated clay with Chitosan using consolidation pressure (**$P$**) of 100, 200, 400, and 800 kPa**

| $P$  (kPa) | $D_{Ch}$ (%) |  |  | Residual | % error |
| --- | --- | --- | --- | --- | --- |
| 100 | 0.5 | 0.038 | 0.040 | -0.0016 | -4.13 |
|  | 1.0 | 0.031 | 0.038 | -0.0070 | -22.39 |
|  | 2.0 | 0.025 | 0.019 | 0.0052 | 21.14 |
|  | 4.0 | 0.076 | 0.073 | 0.0034 | 4.47 |
| 200 | 0.5 | 0.173 | 0.174 | -0.0013 | -0.73 |
|  | 1.0 | 0.171 | 0.151 | 0.0197 | 11.52 |
|  | 2.0 | 0.093 | 0.104 | -0.0118 | -12.73 |
|  | 4.0 | 0.117 | 0.124 | -0.0066 | -5.67 |
| 400 | 0.5 | 0.258 | 0.259 | -0.0010 | -0.41 |
|  | 1.0 | 0.216 | 0.216 | -0.0001 | -0.06 |
|  | 2.0 | 0.128 | 0.136 | -0.0085 | -6.68 |
|  | 4.0 | 0.119 | 0.109 | 0.0097 | 8.17 |
| 800 | 0.5 | 0.239 | 0.235 | 0.0039 | 1.63 |
|  | 1.0 | 0.229 | 0.241 | -0.0125 | -5.48 |
|  | 2.0 | 0.199 | 0.184 | 0.0151 | 7.58 |
|  | 4.0 | 0.147 | 0.154 | -0.0065 | -4.39 |

**Table S9. The comparison of experimental values of primary compression index (****) and best-fit primary compression index (****) of treated clay with Casein using consolidation pressure (**$P$**) of 100, 200, 400, and 800 kPa**

| $P$  (kPa) | $D_{Ca}$ (%) |  |  | Residual | % error |
| --- | --- | --- | --- | --- | --- |
| 100 | 0.5 | 3.576 | 3.585 | -0.009 | -0.26 |
|  | 1.0 | 4.878 | 4.863 | 0.015 | 0.30 |
|  | 2.0 | 7.048 | 7.061 | -0.013 | -0.19 |
|  | 4.0 | 4.867 | 4.859 | 0.008 | 0.16 |
| 200 | 0.5 | 3.460 | 3.454 | 0.005 | 0.16 |
|  | 1.0 | 4.726 | 4.724 | 0.002 | 0.05 |
|  | 2.0 | 6.916 | 6.917 | -0.001 | -0.01 |
|  | 4.0 | 4.753 | 4.760 | -0.007 | -0.15 |
| 400 | 0.5 | 3.290 | 3.295 | -0.005 | -0.16 |
|  | 1.0 | 4.538 | 4.544 | -0.007 | -0.15 |
|  | 2.0 | 6.737 | 6.724 | 0.013 | 0.19 |
|  | 4.0 | 4.647 | 4.648 | -0.001 | -0.02 |
| 800 | 0.5 | 3.136 | 3.127 | 0.009 | 0.28 |
|  | 1.0 | 4.317 | 4.327 | -0.011 | -0.24 |
|  | 2.0 | 6.462 | 6.461 | 0.001 | 0.02 |
|  | 4.0 | 4.507 | 4.507 | 0.001 | 0.01 |

**Table S10. The comparison of experimental values of secondary compression index (****) and best-fit secondary compression index (****) of untreated clay using consolidation pressure (**$P$**) of 100, 200, 400, and 800 kPa**

| $P$  (kPa) |  |  | Residual | % error |
| --- | --- | --- | --- | --- |
| 100 | 0.01206 | 0.01206 | -2.98$\times10^{-6}$ | -0.0247 |
| 200 | 0.01714 | 0.01713 | 9.08$\times10^{-6}$ | 0.0530 |
| 400 | 0.01985 | 0.01986 | -7.88$\times10^{-6}$ | -0.0397 |
| 800 | 0.02070 | 0.02070 | 1.78$\times10^{-6}$ | 0.0086 |

**Table S11. The comparison of experimental values of secondary compression index (****) and best-fit secondary compression index (****) of treated clay with Chitosan using consolidation pressure (**$P$**) of 100, 200, 400, and 800 kPa**

| $P$  (kPa) | $D_{Ch}$ (%) |  |  | Residual | % error |
| --- | --- | --- | --- | --- | --- |
| 100 | 0.5 | 0.01193 | 0.01304 | -0.0011 | -9.32 |
|  | 1.0 | 0.01057 | 0.00936 | 0.0012 | 11.42 |
|  | 2.0 | 0.01042 | 0.00978 | 0.0006 | 6.10 |
|  | 4.0 | 0.00974 | 0.01047 | -0.0007 | -7.51 |
| 200 | 0.5 | 0.01261 | 0.01366 | -0.0010 | -8.30 |
|  | 1.0 | 0.01157 | 0.01038 | 0.0012 | 10.31 |
|  | 2.0 | 0.01028 | 0.01111 | -0.0008 | -8.11 |
|  | 4.0 | 0.01118 | 0.01049 | 0.0007 | 6.16 |
| 400 | 0.5 | 0.01609 | 0.01487 | 0.0012 | 7.56 |
|  | 1.0 | 0.01257 | 0.01245 | 0.0001 | 0.98 |
|  | 2.0 | 0.01204 | 0.01391 | -0.0019 | -15.57 |
|  | 4.0 | 0.01141 | 0.01087 | 0.0005 | 4.69 |
| 800 | 0.5 | 0.0197 | 0.01876 | 0.0009 | 4.78 |
|  | 1.0 | 0.01571 | 0.01823 | -0.0025 | -16.06 |
|  | 2.0 | 0.02364 | 0.02157 | 0.0021 | 8.77 |
|  | 4.0 | 0.01401 | 0.01450 | -0.0005 | -3.51 |

**Table S12. The comparison of experimental values of secondary compression index (****) and best-fit secondary compression index (****) of treated clay with Casein gum using consolidation pressure (**$P$**) of 100, 200, 400, and 800 kPa**

| $P$  (kPa) | $D_{Ca}$ (%) |  |  | Residual | % error |
| --- | --- | --- | --- | --- | --- |
| 100 | 0.5 | 0.0047 | 0.00465 | 0.000048 | 1.01 |
|  | 1.0 | 0.0046 | 0.00448 | 0.000118 | 2.56 |
|  | 2.0 | 0.0044 | 0.00460 | -0.000203 | -4.60 |
|  | 4.0 | 0.0024 | 0.00236 | 0.000037 | 1.55 |
| 200 | 0.5 | 0.0111 | 0.01160 | -0.000496 | -4.47 |
|  | 1.0 | 0.0107 | 0.01067 | 0.000032 | 0.30 |
|  | 2.0 | 0.0106 | 0.01018 | 0.000419 | 3.95 |
|  | 4.0 | 0.0104 | 0.01035 | 0.000045 | 0.43 |
| 400 | 0.5 | 0.0144 | 0.01383 | 0.000570 | 3.96 |
|  | 1.0 | 0.0123 | 0.01220 | 0.000104 | 0.84 |
|  | 2.0 | 0.0104 | 0.01075 | -0.000351 | -3.37 |
|  | 4.0 | 0.0105 | 0.01082 | -0.000323 | -3.08 |
| 800 | 0.5 | 0.015 | 0.01512 | -0.000122 | -0.81 |
|  | 1.0 | 0.0128 | 0.01305 | -0.000253 | -1.98 |
|  | 2.0 | 0.0111 | 0.01097 | 0.000135 | 1.21 |
|  | 4.0 | 0.0109 | 0.01066 | 0.000241 | 2.21 |
